# Coupling Hydrological Scenarios and Water Footprints for Multi-Objective Cropping-Structure Optimization in the Middle and Lower Shiyang River Basin

**DOI:** 10.3390/plants15182863

**Published:** 2026-09-19

**Authors:** Yuhong Pu, Guangping Qi, Yubin Zhang, Yanxia Kang, Yayu Wang, Zhongxia Tang, Haohao Dang, Fusen Yang, Demin Fu

**Affiliations:** 1 College of Water Conservancy and Hydropower Engineering, Gansu Agricultural University, Lanzhou 730070, China; 1073325120974@st.gsau.edu.cn (Y.P.); wangyy@gsau.edu.cn (Y.W.); 1073325120995@st.gsau.edu.cn (H.D.); 1073325120992@st.gsau.edu.cn (F.Y.); 1073325121001@st.gsau.edu.cn (D.F.); 2 Gansu Academy for Water Conservancy, Lanzhou 730000, China; shimano3300@126.com (Y.Z.); 18215129062@163.com (Z.T.)

**Keywords:** cropping-structure optimization, hydrological fluctuations, water footprint, NSGA-III, Shiyang River Basin

## Abstract

Hydrological fluctuations affect cropping-structure optimization in arid inland river basins by altering agricultural water-supply boundaries and crop yields. This study focused on five counties and districts in the middle and lower reaches of the Shiyang River Basin. Based on the 1995–2024 runoff series, four hydrological scenarios—wet, normal, dry, and extremely dry—were constructed. Crop yield responses were incorporated into blue, green, and gray water footprint accounting, and NSGA-III coupled with entropy-weighted TOPSIS was applied for multi-objective optimization. The results showed that (1) under the current cropping structure, unit water footprints generally increased as hydrological conditions shifted from wet to extremely dry, with gray water footprints showing greater sensitivity to yield reductions; (2) the overall directions of cropping-structure adjustment were broadly consistent across scenarios, with the planted areas of wheat, maize, and tubers decreasing by nearly 30% and melon area increasing by nearly 30%, while vegetable and oilseed areas exhibited clear scenario-dependent adjustments; (3) relative to the corresponding 2024 cropping structure evaluated under the same hydrological conditions, the selected compromise schemes increased net economic return by 14.58–16.23 × 10^8^ CNY across the four scenarios. However, the attainable net economic return declined from 73.74 × 10^8^ CNY under the wet scenario to 17.95 × 10^8^ CNY under the extremely dry scenario. The blue water footprint decreased by 18.18–19.07%, whereas the gray water footprint decreased by approximately 17–18% across scenarios. Deteriorating hydrological conditions reduced the attainable economic return and highlighted clear county-level differences in adjustment pathways and economic vulnerability. These findings provide a quantitative basis for scenario- and county-specific cropping-structure optimization and water-resource regulation in arid inland river basins.

## 1. Introduction

Water and land resources are fundamental to food security and regional socioeconomic development [1]. According to the United Nations World Water Development Report 2023, approximately 2.2–3.2 billion people, corresponding to 32–46% of the global population, lived under water stress for at least one month per year, while agricultural withdrawals accounted for approximately 72% of total global water withdrawals [2]. Accordingly, the allocation and efficient use of agricultural water are critical to regional water security [3]. In arid and semi-arid oasis regions, scarce precipitation, strong evaporation, and pronounced fluctuations in surface-water inflow impose substantial constraints on agricultural production and crop distribution. Limited water resources also play a critical role in maintaining oasis ecosystem stability [4]. As water-resource constraints and agricultural environmental pressures continue to intensify, improving water–land resource use efficiency and reducing the environmental costs of agricultural production while maintaining agricultural supply have become key challenges for sustainable agriculture [5].

Cropping-structure optimization is an important approach for coordinating agricultural production, resource consumption, and environmental protection [6,7,8]. Previous studies have extensively explored agricultural production regulation under water-resource constraints. In crop water-requirement assessments, reference crop evapotranspiration, crop coefficients, and effective precipitation are commonly used to quantify crop water consumption and water demand across crops and growth stages [9,10]. In irrigation management, deficit irrigation, regulated deficit irrigation, and irrigation scheduling under different water-supply conditions have been applied to balance crop yield and water-use efficiency [3,11,12]. At the regional scale, linear programming, stochastic programming, and multi-objective evolutionary algorithms have been widely used to optimize crop planting areas and agricultural water allocation while balancing economic returns, food security, water-use constraints, and ecological protection [6]. Meanwhile, the water footprint framework provides an integrated means of characterizing green water consumption, blue water use, and gray water environmental pressure. It has been used to identify high-water-consumption and high-pollution components of agricultural production and has increasingly been incorporated into multi-objective cropping-structure optimization frameworks [13,14,15]. Water footprint assessment has also been widely applied to reveal spatiotemporal variations in agricultural water use and their socioeconomic drivers [16]. In the Shiyang River Basin, previous studies have already demonstrated the value of cropping-structure optimization and water-saving management. Chen et al. [1], for example, developed a credibility-based interval multi-objective crop-area planning model for Minqin County that coordinated agricultural and ecological objectives under interval and fuzzy uncertainty, while Li et al. [17] conducted multi-objective planting-structure optimization at the basin scale. Building on these studies, the present work focuses specifically on how long-term hydrological variability propagates through agricultural water-supply boundaries, crop yield responses, and blue–green–gray water footprints, and it further resolves the resulting optimization responses at the county level. Despite these methodological advances, integration among these components remains limited. Regional optimization models often rely on current or average water-supply conditions, and the effects of long-term wet–dry hydrological variability on agricultural water-supply boundaries have not been fully characterized [18]. Hydrological change, crop yield response, and resource–environmental effects are also frequently treated separately. Moreover, most previous studies emphasize overall regional benefits, whereas inter-county differences in resource pressure, economic risk, and environmental load under extreme water scarcity have received comparatively little attention [19].

The middle and lower reaches of the Shiyang River Basin represent a typical oasis agricultural region in an arid inland river basin, where agricultural water supply is influenced by fluctuations in surface runoff while also being subject to rigid constraints imposed by the groundwater extraction red line [20,21]. Accordingly, this study treats groundwater as a relatively stable policy boundary and considers variations in surface runoff as the primary source of fluctuations in agricultural water availability. On this basis, an analytical framework integrating long-term hydrological scenarios, crop yield responses, blue–green–gray water footprints accounting, and multi-objective optimization was developed for cropping-structure analysis. Specifically, the study aims to: (1) identify wet, normal, dry, and extremely dry hydrological scenarios based on the 1995–2024 runoff series and the Pearson type III distribution, and calibrate the corresponding water regulation coefficient to characterize changes in agricultural water-supply boundaries under different hydrological conditions; (2) quantify the blue, green, and nitrogen-related gray water footprints of different crops across counties under each hydrological scenario, and assess the effects of hydrological fluctuations on crop water footprints as well as county-level resource consumption and environmental pressures; and (3) optimize cropping structure by maximizing net economic return while minimizing blue water consumption and gray water footprint, subject to constraints on water resources, cultivated land area, and food security. NSGA-III and entropy-weighted TOPSIS were then employed to identify optimal compromise schemes and reveal county-level cropping-structure adjustments and their economic–resource–environmental effects under different hydrological scenarios. The resulting framework provides a quantitative basis for water-constrained agricultural land use and production planning and pollution-constrained land management in arid inland river basins.

## 2. Results

### 2.1. Hydrological Scenario Characteristics

#### 2.1.1. Runoff Frequency Characteristics

**Figure 1:**
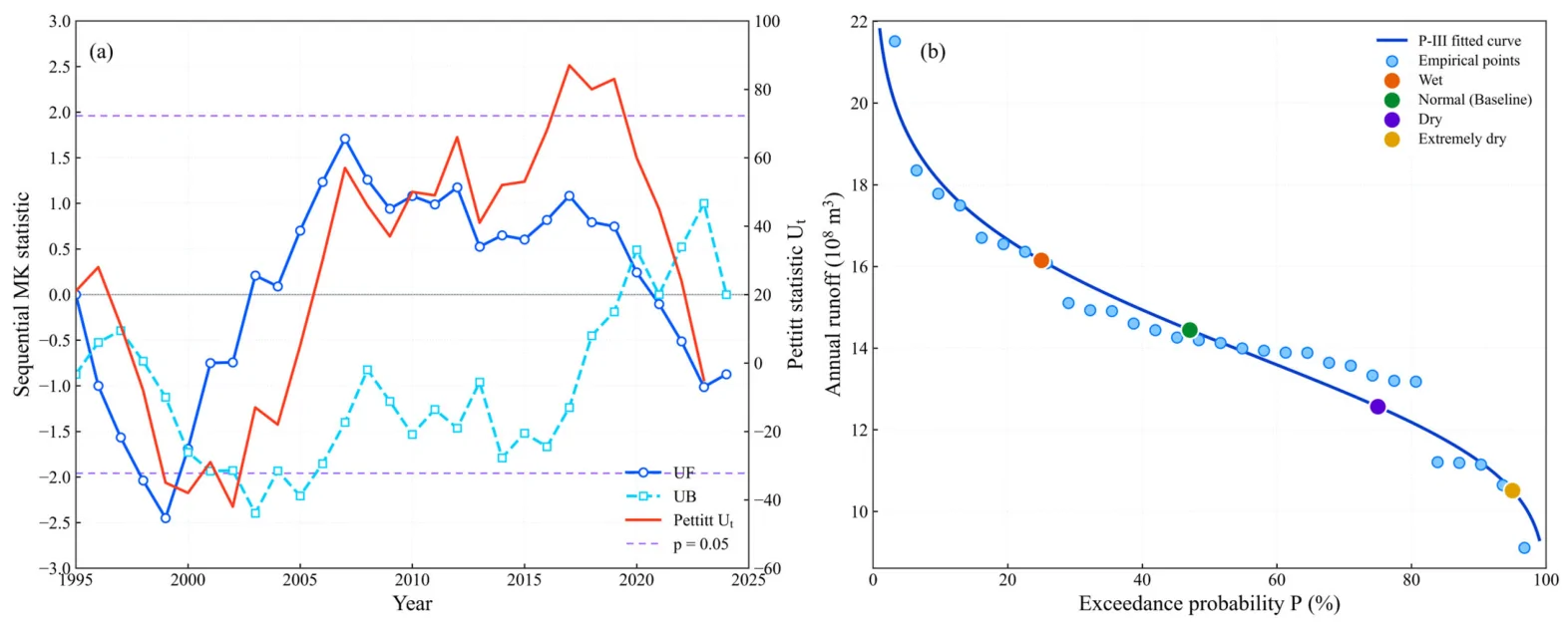
Hydrological diagnosis and runoff scenario calibration during 1995–2024. (**a**) Sequential Mann–Kendall and Pettitt test statistics; (**b**) P-III frequency fitting curve and calibrated hydrological scenario points.

To determine the basis for hydrological scenario classification, trend, change-point, and frequency analyses were conducted for the annual runoff series of the Shiyang River Basin from 1995 to 2024 (Figure 1). The Mann–Kendall test showed no significant trend in the runoff series (Z = −0.856, *p* = 0.392), and the Pettitt test detected no significant change point (*p* = 0.393). These results support the use of conventional frequency analysis for the runoff series. The multi-year average runoff from 1995 to 2024 was 14.44 × 10^8^ m^3^. The P-III frequency curve was then fitted to the runoff series. The fitted parameters were C_v_ = 0.1862 and C_s_/C_v_ = 2.7929. The fitted curve yielded an RMSE of 0.5632 × 10^8^ m^3^, an MAE of 0.4313 × 10^8^ m^3^, and an R^2^ of 0.9484. These metrics indicated a good fit and supported the use of the fitted curve for hydrological scenario calibration.

#### 2.1.2. Scenario Parameter Calibration

The observed runoff in 2024 was 14.44 × 10^8^ m^3^, which was nearly identical to the 1995–2024 multi-year mean of 14.444 × 10^8^ m^3^ (a difference of approximately −0.03%). According to the fitted P-III distribution, the 2024 runoff corresponds to an exceedance probability of approximately 46.5%, which is close to, but not identical to, the theoretical median condition (P = 50%; Q_50_ ≈ 14.21 × 10^8^ m^3^). Therefore, 2024 was retained as the observed normal baseline and assigned θ = 1.00 because it simultaneously represents an approximately normal hydrological condition and provides internally consistent baseline information on the actual cropping structure, crop prices, production costs, and management conditions. In contrast, the wet, dry, and extremely dry scenarios were defined using the theoretical P-III quantiles at P = 25%, 75%, and 95%, respectively, to perturb the hydrological boundary around this observed baseline (Table 1). Thus, the normal scenario serves as an observed reference condition rather than a theoretical P = 50% design year.

### 2.2. Water Footprint Response Under the Current Cropping Structure

#### 2.2.1. Water Footprint Intensity and Scale Characteristics

**Figure 2:**
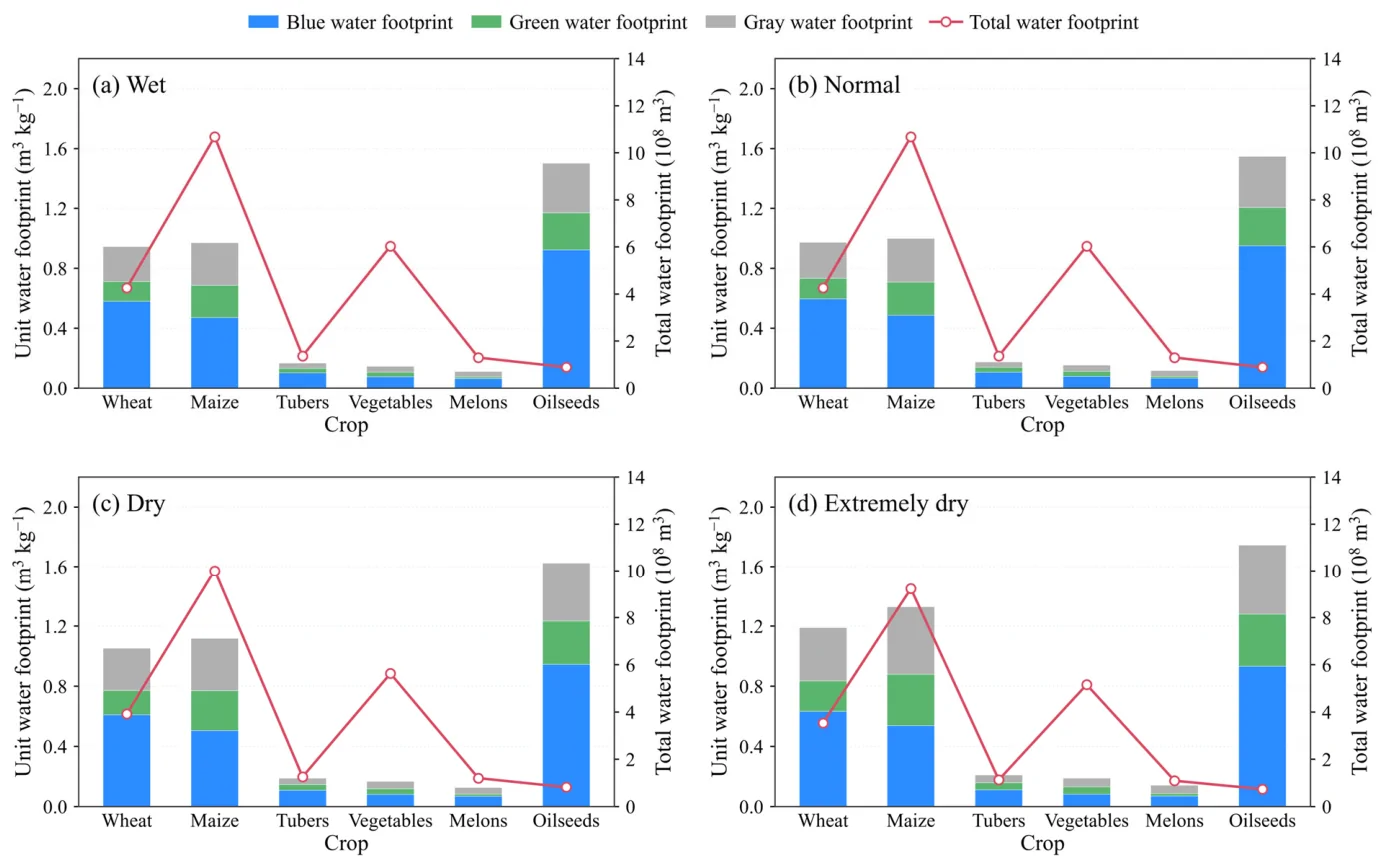
Unit water footprints (UWF) and total water footprints (TWF) of major crops under four hydrological scenarios in the middle–lower reaches of the Shiyang River Basin.

Under all hydrological scenarios, the unit water footprints of all six crop types were dominated by the blue water component. The ranking among crops remained stable: oilseeds > maize > wheat > tubers > vegetables > melons (Figure 2). Under the normal (baseline) scenario, oilseeds had the highest unit water footprint at 1.548 m^3^ kg^−1^. This value was approximately 13.3 times that of melons, which had the lowest unit water footprint (0.117 m^3^ kg^−1^). The unit water footprints of maize and wheat were 1.000 and 0.973 m^3^ kg^−1^, respectively, indicating relatively high water footprint intensities among the grain crops. In contrast, the values for tubers and vegetables were lower at 0.175 and 0.153 m^3^ kg^−1^, respectively.

As hydrological conditions shifted from wet to extremely dry, the unit water footprints of all six crops increased. Maize showed the largest increase, from 0.971 to 1.328 m^3^ kg^−1^. The unit water footprints of vegetables and melons increased by 29.8% and 27.4%, respectively. Wheat and tubers both increased by approximately 26%. Oilseeds showed a comparatively smaller increase but consistently had the highest unit water footprint. The gray water footprint showed a particularly strong response to hydrological deterioration. For maize and oilseeds, it increased from 0.284 and 0.332 m^3^ kg^−1^ under the wet scenario to 0.450 and 0.466 m^3^ kg^−1^ under the extremely dry scenario, respectively. This increase was primarily yield-driven; under fixed nitrogen application and pollutant-related parameters, worsening hydrological conditions reduced crop yields, thereby increasing the gray water footprint per unit of crop production.

In terms of total water footprint, the crop ranking changed to maize > vegetables > wheat > tubers > melons > oilseeds. Under the normal (baseline) scenario, the total water footprint of maize reached 10.683 × 10^8^ m^3^. The total water footprint of vegetables reached 6.036 × 10^8^ m^3^ because of their large planting area, while the total water footprint of wheat was 4.263 × 10^8^ m^3^. The total water footprints of tubers and melons were both approximately 1.3 × 10^8^ m^3^. In contrast, oilseeds accounted for only 0.889 × 10^8^ m^3^ because of their relatively small planting area. These results indicated that hydrological conditions mainly affected water footprint intensity per unit of crop production, whereas crop planting area largely determined the basin-scale pattern of total water footprints.

#### 2.2.2. County-Level Differences in Water Footprints Under Different Hydrological Scenarios

**Figure 3:**
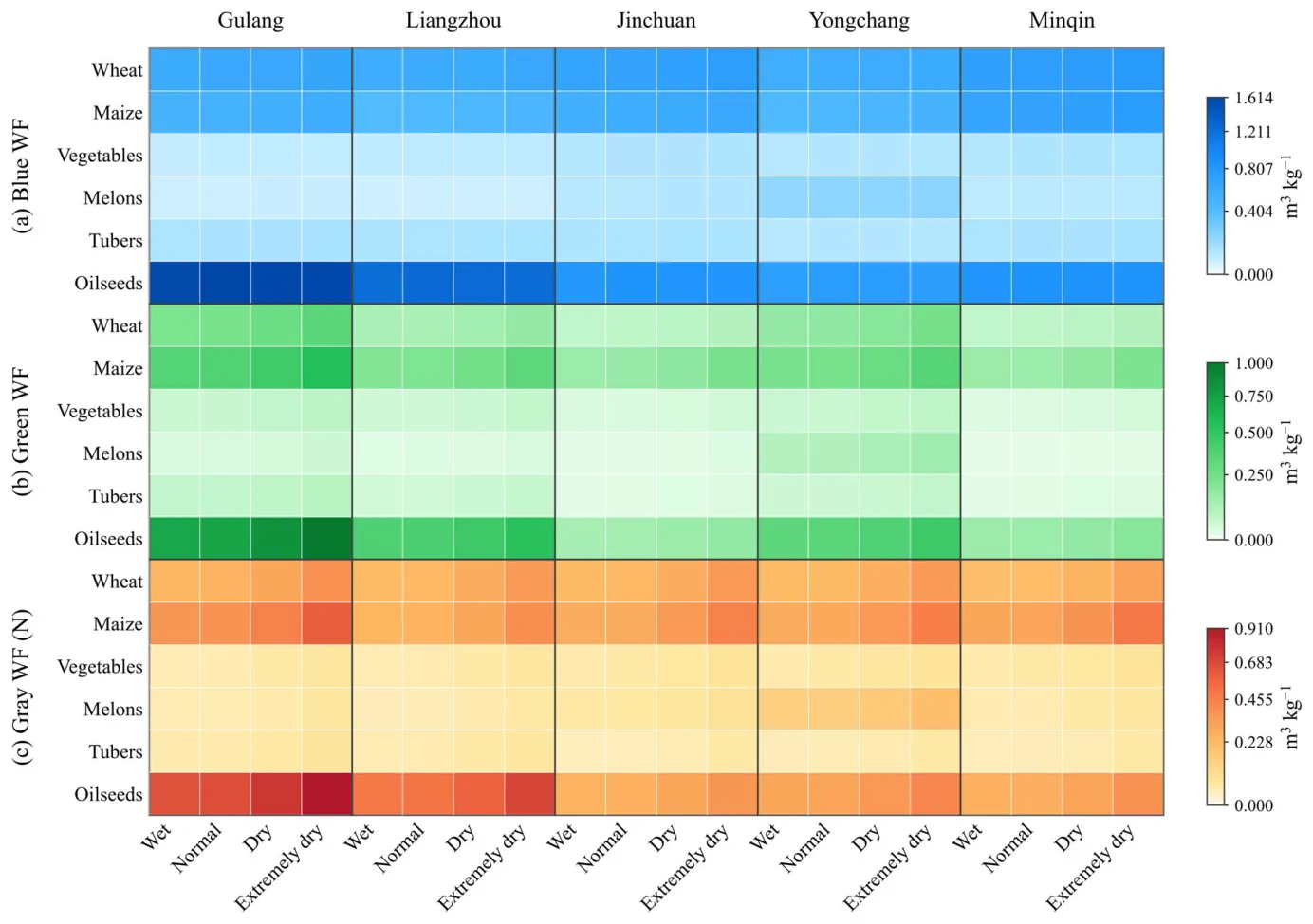
County-level variations in crop unit water footprints under different hydrological scenarios.

At the county scale, crop water footprints showed clear spatial differences. County-level differences in blue and green water footprints were generally small, although some crops exhibited pronounced local hotspots (Figure 3). Under the normal (baseline) scenario, the blue water footprint of wheat ranged from 0.564 to 0.722 m^3^ kg^−1^ across counties, indicating relatively low spatial variation. In contrast, the blue water footprint of oilseeds in Gulang County reached 1.614 m^3^ kg^−1^. This value was 2.15 times that in Yongchang County (0.750 m^3^ kg^−1^).

Spatial differences were more pronounced for the gray water footprint. These differences mainly reflected variations in crop yield among counties under the same nitrogen application parameters. The gray water footprint of oilseeds in Gulang County was 0.668 m^3^ kg^−1^, compared with 0.509 m^3^ kg^−1^ in Liangzhou District and 0.272 m^3^ kg^−1^ in Jinchuan District. The maximum-to-minimum ratio across counties was 2.46. The gray water footprint of maize in Gulang County was 0.382 m^3^ kg^−1^, which was 48.1% higher than that in Liangzhou District. These results indicated relatively high gray water environmental loads per unit of crop production in Gulang County.

As hydrological conditions shifted from wet to extremely dry, declining crop yields generally increased unit water footprints. The increase was particularly evident for the gray water footprint. The gray water footprint of oilseeds in Gulang County increased from 0.649 to 0.910 m^3^ kg^−1^, which is an increase of 40.2%. For maize in Liangzhou District, it increased from 0.251 to 0.397 m^3^ kg^−1^, representing an increase of 58.2%. For wheat in Jinchuan District, it increased from 0.236 to 0.359 m^3^ kg^−1^, an increase of 52.1%. This pattern mainly resulted from relatively fixed nitrogen inputs being distributed over lower crop yields. This indicated that drought-induced yield reductions could increase the potential nitrogen-related environmental load per unit of crop production. Areas with initially high gray water footprints therefore remained at relatively high levels under the extremely dry scenario. Detailed county- and crop-specific blue, green, and gray unit water footprints under all hydrological scenarios are provided in Supplementary Tables S1–S3.

### 2.3. Cropping-Structure Optimization Results Under Different Hydrological Scenarios

#### 2.3.1. Pareto Fronts and Objective Trade-Offs

**Figure 4:**
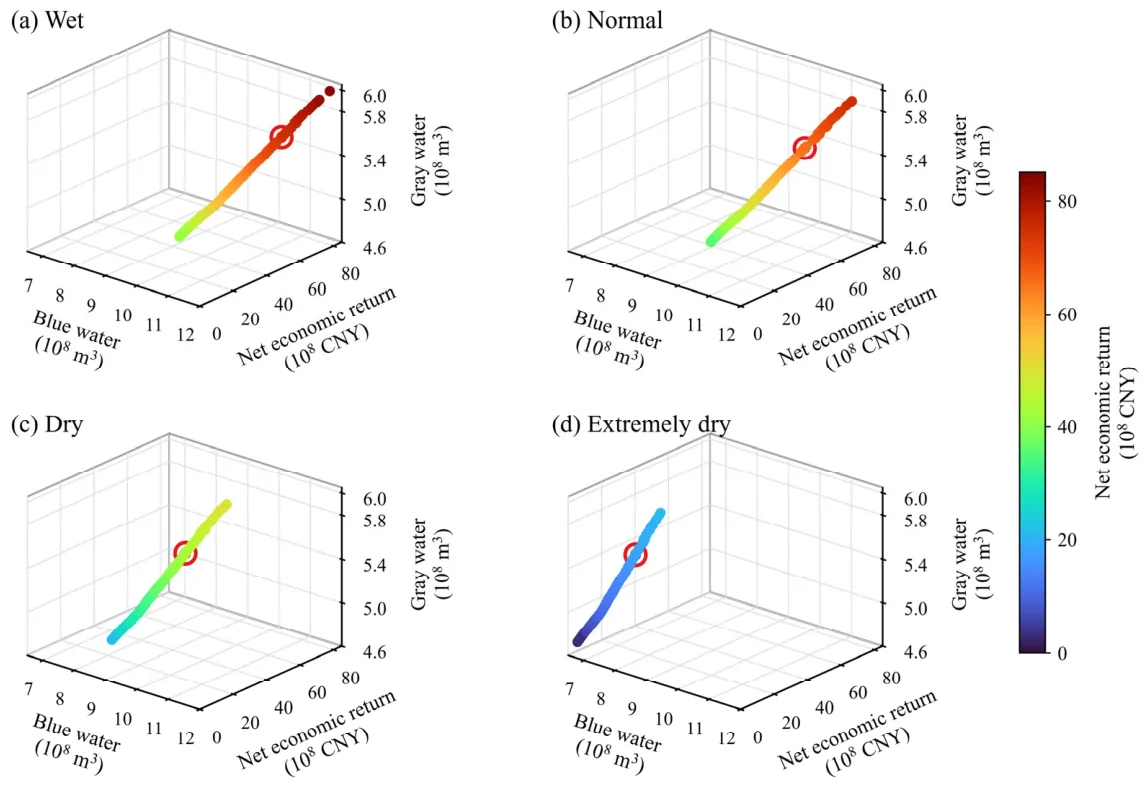
Pareto front distributions under four hydrological scenarios in the Shiyang River Basin.

Pareto non-dominated solution sets were obtained under all hydrological scenarios (Figure 4). Across the five independent runs, the feasible solution ratio was 1.00 for all scenarios. The normalized hypervolume ranged from 0.5148 to 0.5718, with coefficients of variation below 0.5%. The Spacing metric ranged from 0.0045 to 0.0055. These metrics indicate stable and repeatable search performance across the five independent NSGA-III runs and a relatively uniform distribution of the obtained non-dominated solutions. They were used to assess run-to-run stability and solution distribution rather than to independently verify convergence to the exact Pareto front. To independently evaluate solution accuracy, the selected NSGA-III compromise solutions were further benchmarked against an ε-constraint linear programming approach under the same model constraints. The relative optimality gaps were 0.018%, 0.171%, 0.062%, and 0.176% under the wet, normal, dry, and extremely dry scenarios, respectively, indicating close agreement between the NSGA-III and linear programming solutions.

Across all four scenarios, the Pareto solution sets formed a nearly one-dimensional trade-off curve, reflecting strong associations among the three objectives despite their distinct management meanings. Solutions with higher net economic return generally required greater blue water consumption and were accompanied by higher gray water footprints. As hydrological conditions shifted from wet to dry and extremely dry, the attainable range of net economic return narrowed markedly, and the Pareto solution sets shifted toward lower net economic return and lower blue water consumption. This pattern indicated that stronger water-supply constraints reduced the feasible space for cropping-structure adjustment and strengthened the economic–resource trade-off. Based on the entropy weights shown in Table 2, entropy-weighted TOPSIS was used to identify the compromise solution with the highest relative closeness coefficient within each non-dominated solution set under each scenario. These solutions are marked by red points in Figure 4. Detailed entropy weights and the objective values of the selected compromise solutions are provided in Supplementary Table S4.

#### 2.3.2. Optimal Compromise Schemes Under Different Hydrological Scenarios

**Figure 5:**
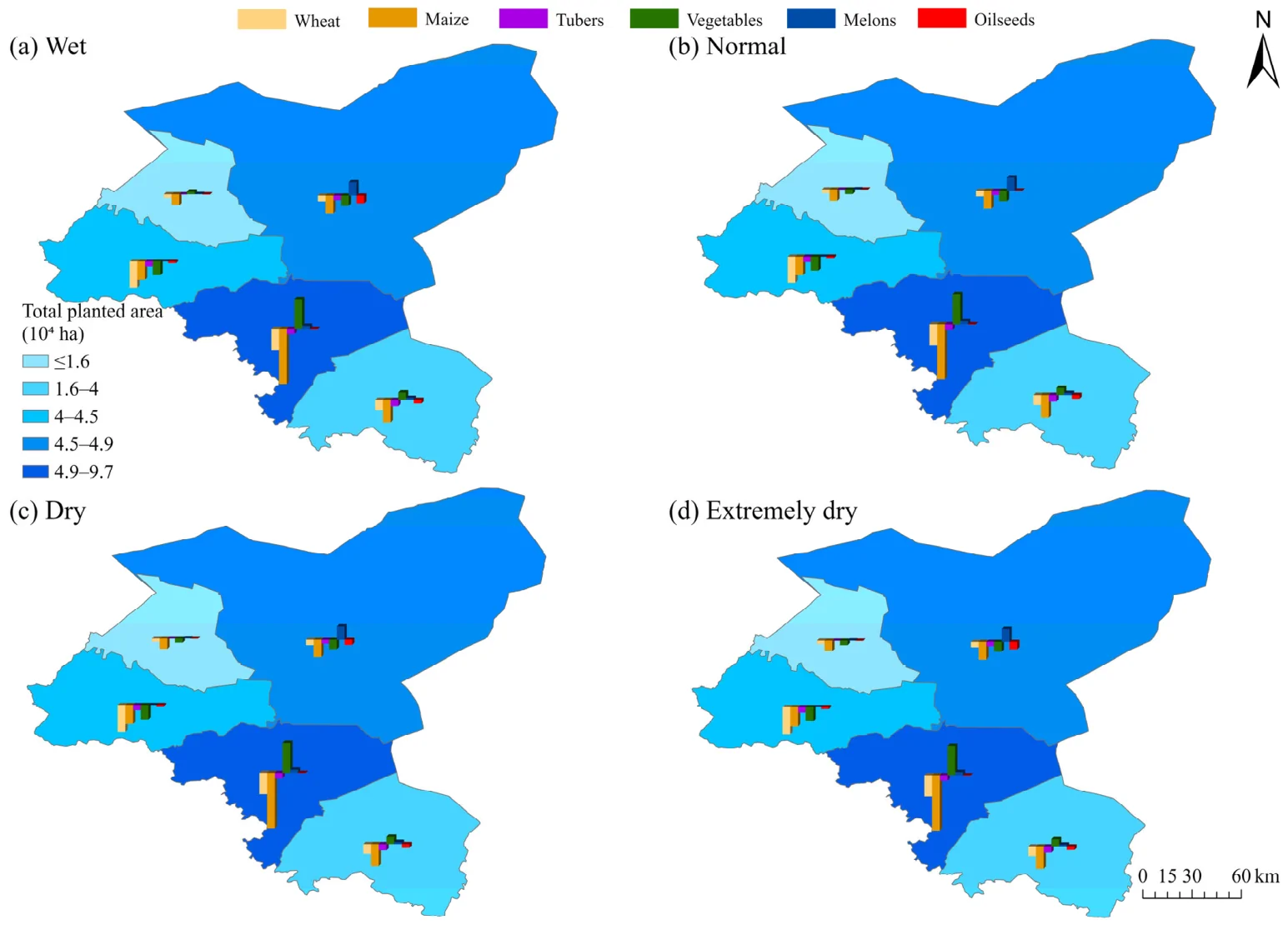
Spatial patterns of county-level crop area adjustments under different hydrological scenarios. Background shading denotes the optimized total planted area in each county using a common classification across all four scenarios, while colored bars qualitatively indicate the direction and relative magnitude of crop-specific planting-area changes relative to the 2024 baseline.

The multi-objective optimization results showed that the overall direction of cropping-structure adjustment under the optimal compromise schemes was broadly consistent across hydrological scenarios, although some crops exhibited clear scenario-dependent responses (Figure 5). Relative to the 2024 status quo, the areas planted with wheat, maize, and tubers were reduced by nearly 30% under all scenarios, approaching their lower adjustment bounds, whereas melon area consistently increased by nearly 30%. Vegetable area increased by 7.02% under the wet scenario and by 3.56–3.84% under the normal, dry, and extremely dry scenarios. Oilseed crops showed an overall contraction across all scenarios, but the magnitude of reduction gradually narrowed from 29.94% under the wet scenario to 11.16% under the extremely dry scenario. Overall, the optimized cropping structure was characterized by stable reductions in grain crops and tubers, persistent expansion of melons, and flexible adjustments in vegetables and oilseeds in response to changing hydrological conditions.

Cropping-structure optimization further altered regional net economic return and total water footprint volumes (Table 3). When the 2024 cropping structure was evaluated under the corresponding hydrological conditions, its net economic returns were 57.51, 50.63, 28.55, and 3.08 × 10^8^ CNY under the wet, normal, dry, and extremely dry scenarios, respectively. The corresponding optimized schemes achieved 73.74, 65.21, 43.40, and 17.95 × 10^8^ CNY, indicating that cropping-structure adjustment improved net economic return under all four scenarios. Meanwhile, the blue and gray water footprints of the optimized schemes were reduced by 18.18–19.07% and 17.31–18.10%, respectively, relative to the corresponding same-scenario 2024-structure baselines. These results indicate that deteriorating hydrological conditions reduced the attainable economic level, while cropping-structure adjustment partially offset these losses. Detailed basin-scale comparisons between the same-scenario 2024 baseline and the optimized schemes are provided in Supplementary Table S5.

#### 2.3.3. County-Level Effects of Cropping-Structure Optimization

Using the 2024 cropping structure as a common baseline, the optimal county-level schemes exhibited pronounced spatial differentiation across hydrological scenarios (Figure 5). Under the wet scenario, Liangzhou District and Gulang County were mainly characterized by reductions in grain-crop areas and expansion of vegetable and melon areas. Minqin County was primarily characterized by melon expansion accompanied by a contraction in vegetable area, while both vegetables and melons expanded in Jinchuan District. In contrast, Yongchang County was generally characterized by reductions in crop areas. Under the normal scenario, the dominant adjustment patterns in most counties were broadly maintained, although vegetable area in Jinchuan District shifted from an increase of 17.68% under the wet scenario to a decrease of 29.91%. Under the dry and extremely dry scenarios, the main county-level optimization pathways remained relatively stable: Liangzhou District and Gulang County continued to be dominated by the expansion of vegetables and melons, Minqin County and Jinchuan District showed pronounced expansion of melons, whereas the scope for adjustment in Yongchang County was relatively limited. The complete optimized planting areas and corresponding percentage changes for each county and crop under the four hydrological scenarios are provided in Supplementary Tables S6 and S7.

**Figure 6:**
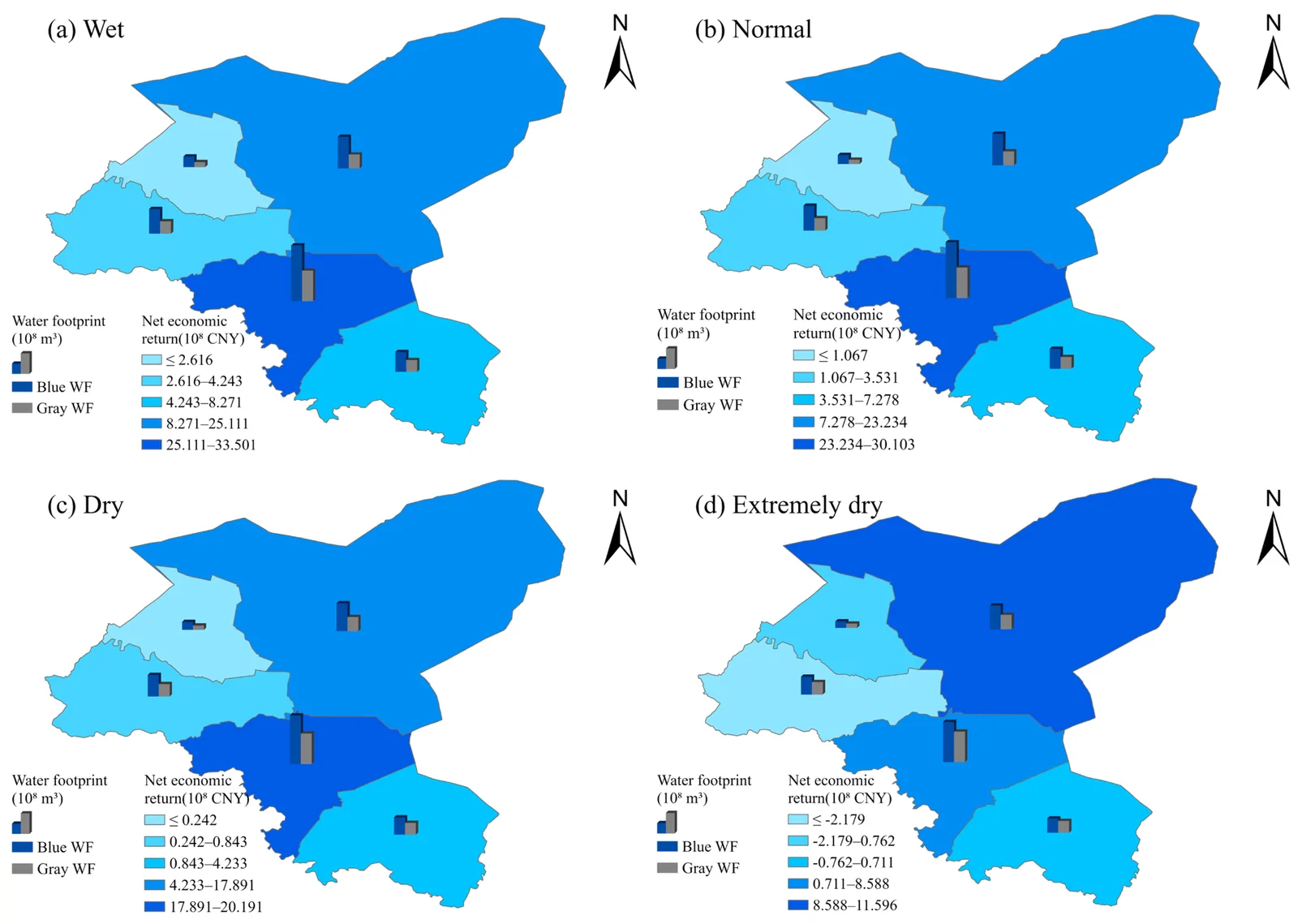
County-level net economic return and water footprint responses under different hydrological scenarios. Background classifications of net economic return were determined separately for each hydrological scenario to preserve within-scenario spatial contrasts; therefore, colors should not be interpreted as directly comparable absolute values across panels. The superimposed bars qualitatively indicate the relative magnitudes of blue and gray water footprints.

County-level responses in net economic return and water footprints also differed markedly among hydrological scenarios (Figure 6). As hydrological conditions shifted from wet to extremely dry, the optimized blue water footprint decreased substantially in all counties. In Liangzhou District, it declined from 4.18 × 10^8^ m^3^ to 3.01 × 10^8^ m^3^, while in Minqin County it decreased from 2.37 × 10^8^ m^3^ to 1.78 × 10^8^ m^3^. By contrast, the gray water footprint showed relatively limited variation overall, remaining at approximately 2.29 × 10^8^ m^3^, 0.88 × 10^8^ m^3^, and 0.93 × 10^8^ m^3^ in Liangzhou District, Gulang County, and Yongchang County, respectively. Meanwhile, net economic return generally declined across counties, falling to −0.76 × 10^8^ CNY in Jinchuan District and −2.18 × 10^8^ CNY in Yongchang County under the extremely dry scenario. Overall, increasingly stringent water-supply constraints primarily reduced county-level blue water consumption and net economic return, whereas the spatial pattern of the gray water footprint remained comparatively stable. Detailed county-level comparisons between the same-scenario 2024 baseline and the optimized schemes are provided in Supplementary Table S8.

#### 2.3.4. Sensitivity Analysis of Optimization Results

**Figure 7:**
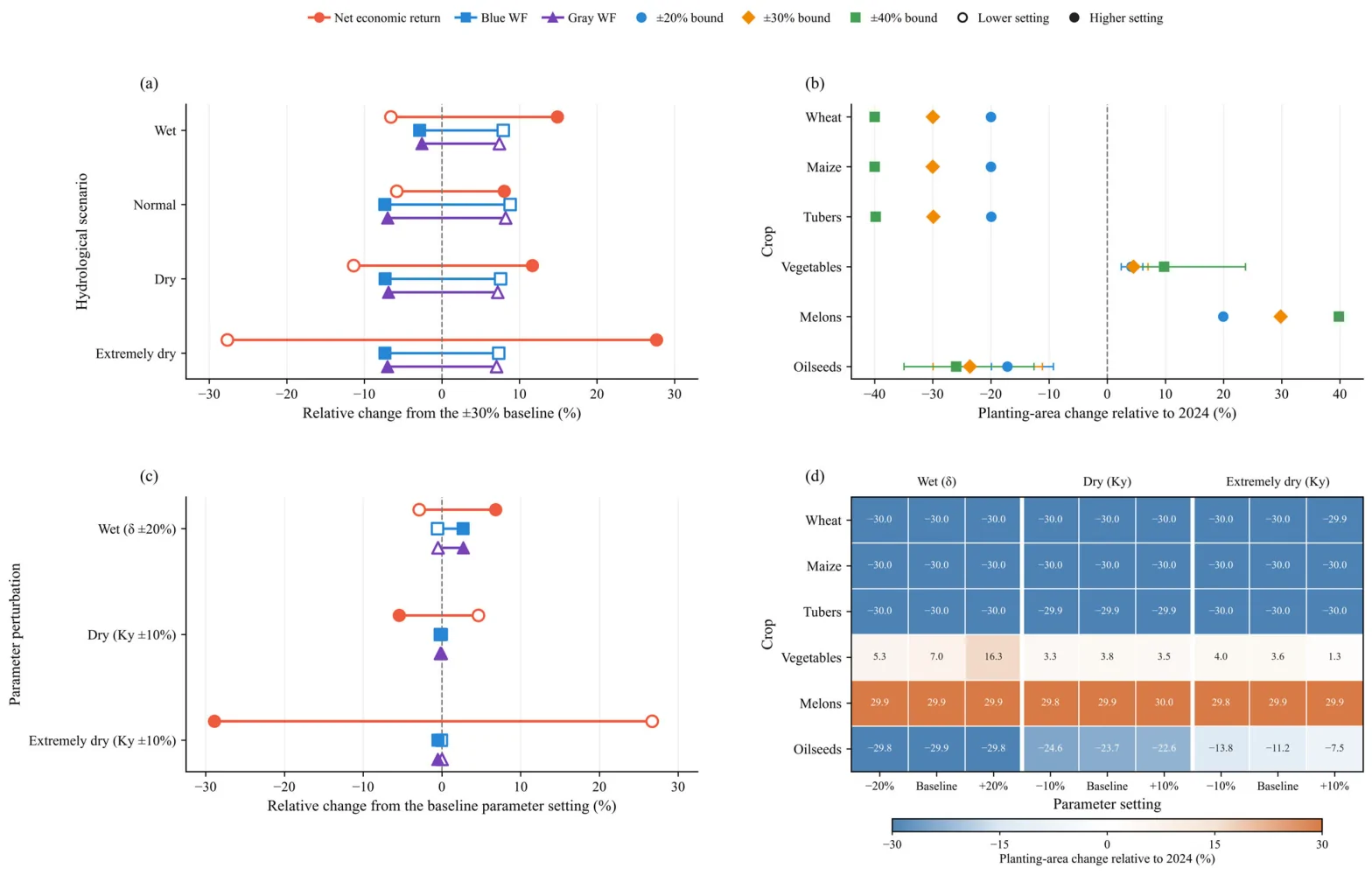
Sensitivity analysis of optimization outcomes: (**a**) changes in the three objectives under different crop-area bounds; (**b**) crop-specific area changes under ±20%, ±30%, and ±40% bounds; (**c**) changes in the three objectives under δ and $K_{y}$ perturbations; (**d**) crop-specific area changes under the corresponding parameter settings.

Sensitivity analyses were conducted by varying the crop-area adjustment bounds from the baseline ±30% to ±20% and ±40%, the crop-specific $K_{y}$ values by ±10% under the dry and extremely dry scenarios, and the wet-scenario yield-increase coefficient δ by ±20%, while all other parameters were held constant (Figure 7). Narrowing the crop-area bound to ±20% reduced net economic return by 5.82–27.67%, whereas relaxing it to ±40% increased net economic return by 8.03–27.67% relative to the baseline bound; the corresponding changes in blue and gray water footprints were generally within approximately 9%. Wheat, maize, and tubers remained at or close to their lower adjustment bounds, while melons remained at or close to the upper bound under all three settings, indicating that the stability of these adjustment directions was partly influenced by the imposed area constraints. Under the extremely dry scenario, net economic return ranged from 12.99 to 22.92 × 10^8^ CNY as the adjustment bound increased from ±20% to ±40%. A ±10% variation in $K_{y}$ changed net economic return by approximately −5.43% to +4.64% under the dry scenario and by −28.90% to +26.70% under the extremely dry scenario, while changes in blue and gray water footprints remained below 0.52%. Under the wet scenario, a ±20% variation in δ changed the net economic return by −2.88% to +6.83%, with blue and gray water footprint changes remaining within approximately 2.71%. Overall, the principal crop-adjustment directions remained consistent across the tested parameter settings, although the magnitude of the economic response became more sensitive under increasingly water-limited conditions.

## 3. Discussion

### 3.1. Hydrological Scenario Decoupling and Water-Supply Boundaries

Agricultural production in the Shiyang River Basin is highly dependent on limited water resources, and hydrological fluctuations primarily affect feasible space for cropping-structure optimization by altering the amount of water available for agriculture. Across the wet, normal, dry, and extremely dry scenarios constructed in this study, the water regulation coefficient (θ) decreased from 1.12 to 0.72, indicating a progressive contraction of the agricultural water-supply boundary as inflow conditions deteriorated. Deteriorating hydrological conditions affected the optimization system through reduced realizable irrigation supply and scenario-dependent crop yields, thereby altering net economic returns and the trade-offs among economic, water-resource, and environmental objectives. Therefore, the substantial reduction in blue water consumption accompanied by a sharp decline in net economic return under the extremely dry scenario does not represent a simple water-saving effect but rather reflects the combined effects of reduced crop productivity, limited irrigation availability, and constrained structural adjustment under severe water scarcity.

This study adopted a hydrological–economic decoupling framework [22], in which socioeconomic conditions such as 2024 prices, production costs, and production practices were held relatively constant, while alternative water-supply scenarios were constructed according to the frequency characteristics of the long-term runoff series. Consequently, differences among scenarios primarily reflected changes in hydrological conditions and agricultural water-supply boundaries. Compared with approaches that directly select representative historical wet and dry years [23], this treatment reduces interference from interannual variations in prices, management practices, and production conditions, thereby allowing the effects of hydrological fluctuations on cropping-structure optimization to be identified more clearly. The results showed that changing hydrological conditions did not alter the overall direction of cropping-structure optimization but substantially affected net economic returns and the intensity of trade-offs among objectives. This suggests that cropping-structure adjustment can still partially alleviate water-resource pressure under normal and moderately dry conditions, whereas under extremely dry conditions, structural optimization alone is insufficient to fully offset the economic risks arising from reduced water availability. Accordingly, cropping-structure regulation in the Shiyang River Basin should be adapted to different levels of water availability, and dynamic management under extremely dry conditions should further integrate water-supply allocation with regional risk regulation.

### 3.2. Crop Water Footprint Response Mechanisms Under Hydrological Change

Crop water footprints in the Shiyang River Basin exhibited clear scale-dependent responses to hydrological change. Under the current cropping structure, the blue water footprint was the dominant component for all six crop types, reflecting the strong dependence of oasis agriculture on irrigation water supply. As hydrological conditions shifted from wet to extremely dry, declining crop yields increased water and environmental burdens per unit of crop output. The blue water response was more moderate because realizable irrigation water use was simultaneously restricted under water-deficit scenarios, whereas the green and gray components were affected mainly through declining crop yield [24]. Under the extremely dry scenario, the unit gray water footprints of maize and oilseeds increased by 58.5% and 40.3%, respectively, relative to the wet scenario. This response arises because crop yield appears in the denominator of the unit gray water footprint. When nitrogen application intensity and pollutant-related parameters remain relatively stable, yield reductions generate a pronounced “yield dilution effect,” thereby increasing the potential environmental burden per unit of agricultural output [25]. Drought therefore affects not only agricultural water availability but also the resource and environmental burdens per unit of agricultural output by reducing production efficiency.

Cropping-structure optimization, by contrast, mainly altered the total regional water footprint through the reallocation of crop areas. After optimization, the total blue water footprint declined progressively as water-supply conditions became more restrictive, whereas the total gray water footprint varied only slightly among scenarios, ranging from 5.47 × 10^8^ m^3^ to 5.52 × 10^8^ m^3^. This difference arises because total blue water consumption is jointly constrained by the agricultural water-supply boundary and crop-specific blue water requirements. In contrast, when nitrogen application intensity per unit area is held constant, the total gray water footprint is determined primarily by crop-area allocation and crop-specific nitrogen inputs. Yield reductions themselves mainly affect the unit gray water footprint and do not directly increase the regional total gray water footprint. Consistently, the nitrogen-application-weighted planted area decreased by 17.31–18.10% across scenarios, exactly matching the corresponding reduction in total gray water footprint. Hydrological change therefore affected water footprints through three distinct pathways: yield reductions increased unit water footprints, tighter water-supply constraints reduced total blue water consumption, and crop-area reallocation regulated the total gray water footprint. Compared with water footprint accounting under a static cropping structure [19,26,27], these findings indicate that agricultural water-resource management in arid regions should simultaneously consider production efficiency per unit output and regional total-load constraints, while coordinating cropping-structure adjustment with water-saving and nitrogen-reduction measures.

### 3.3. County-Specific Regulation Strategies

Cropping-structure optimization can improve agricultural economic returns, resource use efficiency, and environmental performance [6,18]. However, at the basin scale in arid regions, such improvements do not imply that all counties benefit simultaneously; rather, they are accompanied by a spatial restructuring of water-resource constraints, net economic returns, and environmental pressures [28]. Under the combined constraints of food security, blue water availability, and gray water environmental load, distinct adjustment pathways emerged among counties. In Liangzhou District, a major agricultural production area, optimization was mainly characterized by reductions in maize area and expansion of vegetable and melon areas. High-value crops should therefore be preferentially allocated to areas with relatively reliable irrigation supply, while maintaining strict control over total agricultural water use and promoting water-saving irrigation and reduced nitrogen inputs. Gulang County also showed potential for expanding vegetables and melons, but priority should be given to freeing water and land resources by reducing less efficient grain and oilseed crops, together with strengthened nitrogen-input management to prevent the expansion of high-value crops from creating additional pressure on water resources and gray water environmental loads. In Minqin County, the comparative advantage was more strongly reflected in melon expansion, whereas vegetable expansion was more constrained. Accordingly, water-efficient and intensive development of specialty melons should be promoted, while the expansion of water-intensive crops should be approached cautiously under the groundwater extraction red-line constraint [29].

By contrast, Jinchuan District and Yongchang County exhibited greater economic sensitivity to hydrological change. In Yongchang County, net economic return declined from 4.24 × 10^8^ CNY under the wet scenario to −2.18 × 10^8^ CNY under the extremely dry scenario, indicating that cropping-structure adjustment alone is insufficient to fully offset the economic losses caused by reduced water availability and declining crop yields under extreme water scarcity. These negative returns were mainly caused by the lower crop-area bounds and food- security constraints, which limited further area reduction under severe water scarcity. Jinchuan District should therefore enhance the flexibility of its cropping structure and avoid rigid dependence on water-intensive and input-intensive crops. Yongchang County should increase the share of low-water-demand crops and adopt drought-resilient production measures while maintaining basic food production and improve production stability under extreme drought through water-saving irrigation and emergency water-resource allocation. For counties facing substantial economic losses under extremely dry conditions, risk-sharing mechanisms may also be developed through interregional water-resource coordination [30], water-right reallocation [31], and agricultural insurance [32]. Overall, cropping-structure regulation in the Shiyang River Basin should avoid uniform crop-adjustment ratios and instead follow differentiated pathways characterized by intensive development of water-efficient, high-value vegetables and melons in Liangzhou and Gulang, water-efficient development of specialty melons in Minqin, flexible regulation in Jinchuan, and food security-oriented drought-resilient production in Yongchang, thereby balancing basin-wide optimization efficiency with county-level economic stability.

### 3.4. Limitations and Future Directions

Although the proposed framework provides a systematic basis for evaluating cropping-structure adjustment under different hydrological scenarios, several uncertainties should be considered when interpreting the results. Socioeconomic parameters and management conditions were maintained at the 2024 baseline to isolate the effects of hydrological variability. Holding crop prices constant may therefore overestimate drought-induced economic losses, because reduced regional output could exert upward pressure on crop prices. Groundwater was treated as a relatively stable policy-constrained boundary. Actual groundwater abstraction may nevertheless deviate from this policy boundary during severe droughts because of emergency water supply or water-user responses, and such surface-water–groundwater substitution was not explicitly simulated in the present framework. In addition, the water regulation coefficient θ was used as a county-scale proxy for relative water-supply conditions rather than as a direct representation of field-scale crop water stress, and nitrogen application and pollutant-related parameters were assumed to remain relatively stable across scenarios. Although algorithmic randomness was reduced through repeated NSGA-III runs and multiple performance metrics, future studies could further incorporate dynamic socioeconomic responses, adaptive irrigation and fertilizer management, and more detailed crop water-stress processes to improve the representation of long-term agricultural adaptation under hydrological variability.

## 4. Materials and Methods

### 4.1. Study Area

**Figure 8:**
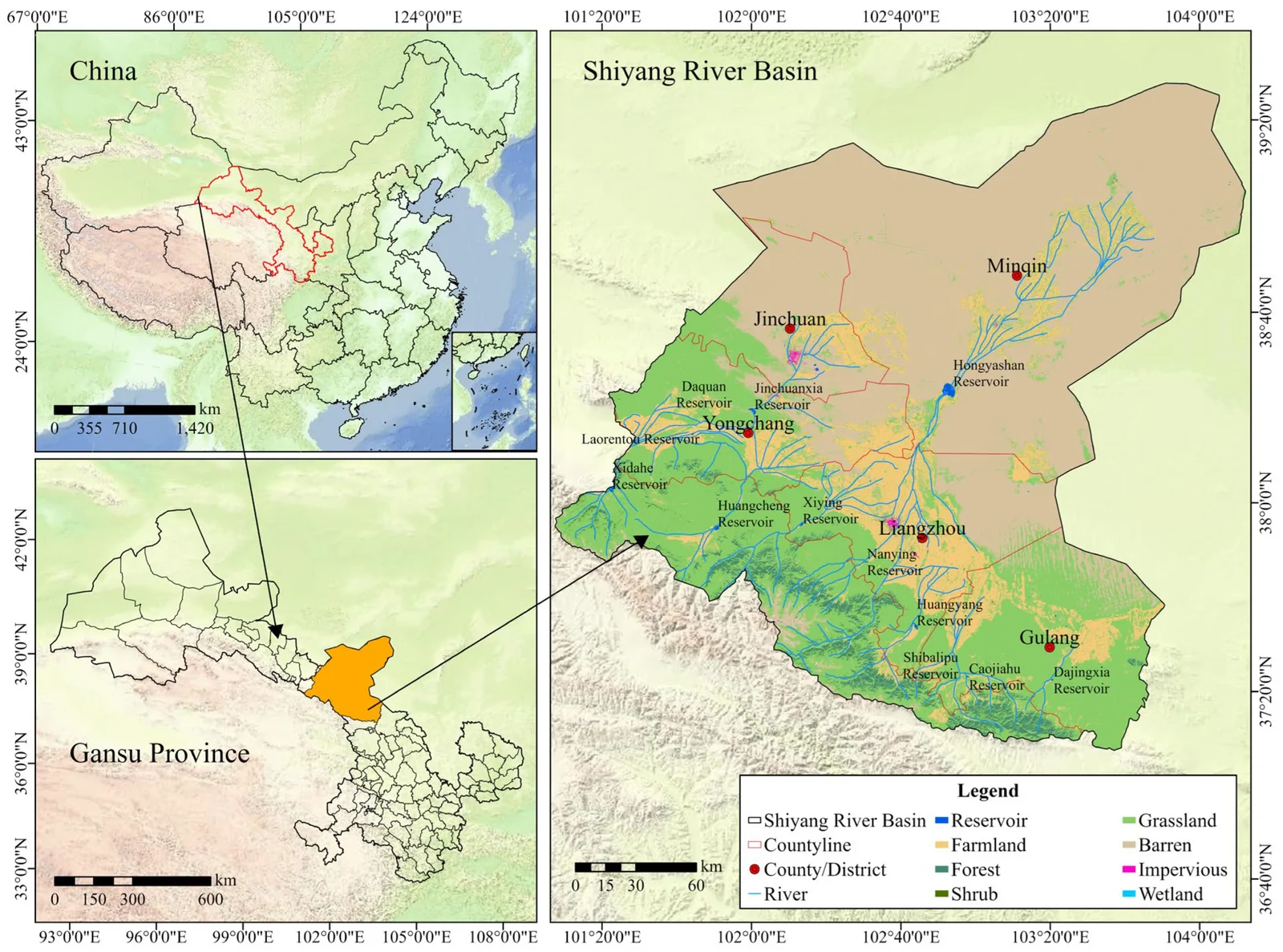
Location, administrative divisions, and land-use characteristics of the study area in the middle and lower reaches of the Shiyang River Basin, Gansu Province, China.

The Shiyang River Basin is located at 36°29′–39°27′ N and 101°41′–104°16′ E. It is a typical arid inland river basin with extensive oasis agriculture [33]. Agricultural irrigation accounts for more than 83.34% of total water use in the basin [34]. The main crops include maize, wheat, vegetables, and melons. Groundwater extraction is strictly controlled under regional water-resource management policies and extraction red lines [35,36]. In this study, Liangzhou District, Minqin County, Gulang County, Yongchang County, and Jinchuan District in the middle and lower reaches of the basin were selected as the study units [17] (Figure 8). These counties encompass the major oasis croplands and agricultural production areas of the basin. They are therefore representative areas for analyzing cropping-structure adjustment and its water-resource and environmental effects under hydrological fluctuations. These administrative units were used as the decision scale because agricultural statistics, water-use controls, and cropping-structure management are implemented primarily at the county or district level. However, this aggregation may mask within-county heterogeneity and does not correspond exactly to hydrological boundaries.

### 4.2. Data Sources

The data used in this study mainly included crop production data, agricultural input data, socioeconomic data, and meteorological and hydrological data. Meteorological data, including precipitation, temperature, relative humidity, wind speed, and sunshine duration, were obtained from the National Meteorological Science Data Center (http://data.cma.cn). Crop coefficients and irrigation parameters were obtained primarily from the FAO database and the Irrigation Water Quota for Major Crops in Northern China [37]. Annual runoff data from 1995 to 2024 were obtained from the Gansu Provincial Water Resources Bulletin. Socioeconomic data, including crop planted area, yield, selling price, production costs, fertilizer application, and population in each county or district, were obtained from the Gansu Statistical Yearbook and the Compilation of Cost–Benefit Data of Agricultural Products in China.

**Figure 9:**
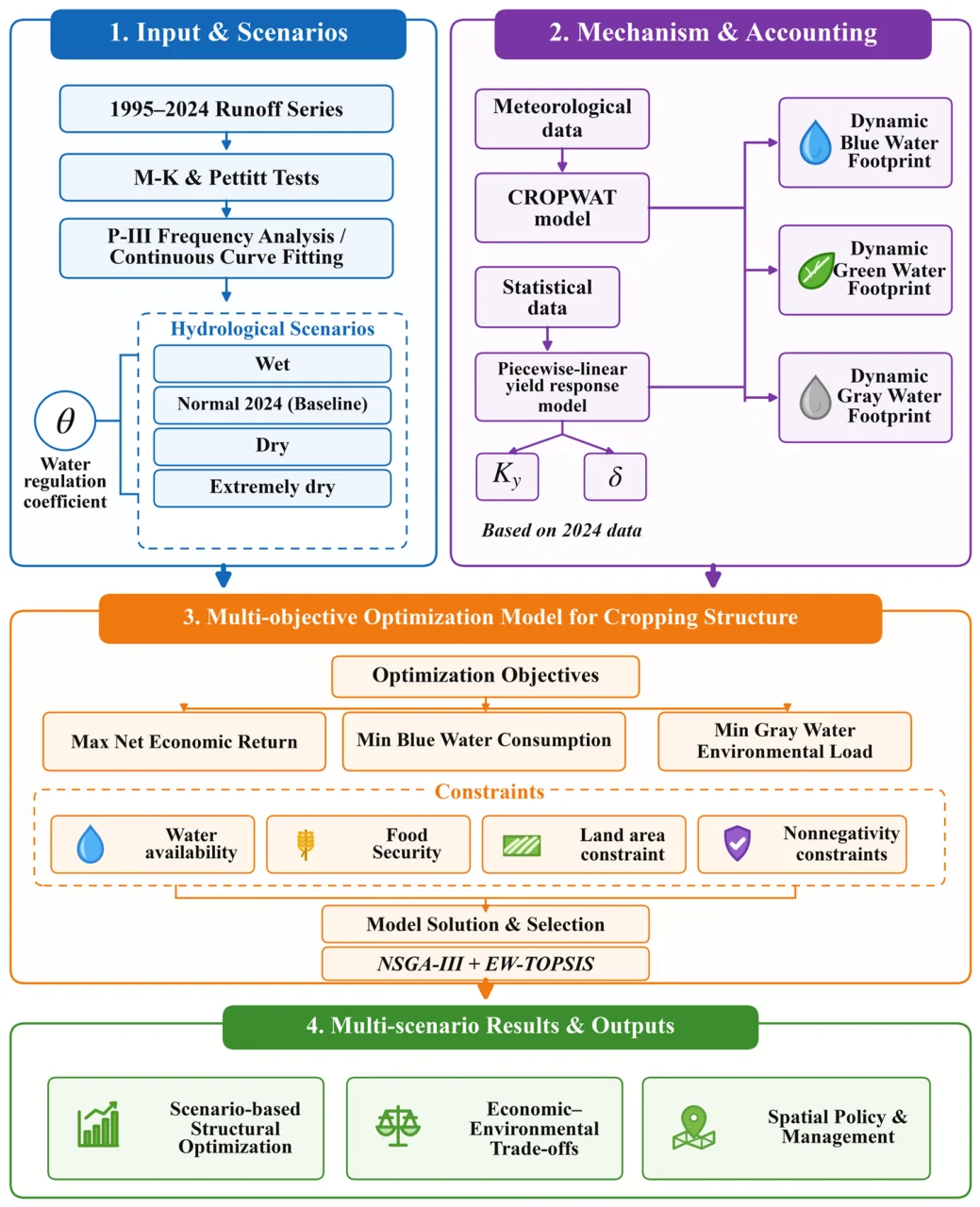
Flowchart of the multi-objective optimization model for cropping structure considering hydrological fluctuations and environmental loads.

To ensure the comparability of optimization results under different hydrological scenarios, 2024 was used as the actual baseline year. The 2024 cropping structure, crop yields, prices, costs, and agricultural water-use constraints were consistently used as baseline inputs for all scenario calculations and comparisons. To systematically organize the analytical workflow for cropping-structure optimization under long-term hydrological fluctuations, this study developed a four-layer integrated framework. The framework included data input and scenario identification, mechanistic linkage and water footprint accounting, multi-objective optimization modeling, and solution screening and result presentation (Figure 9).

### 4.3. Hydrological Scenario Construction

#### 4.3.1. Runoff Frequency Analysis

The Mann–Kendall test was used to identify monotonic trends in the annual runoff series of the Shiyang River Basin from 1995 to 2024, and the Pettitt test was applied to detect potential change points, thereby assessing whether the runoff series exhibited non-stationarity that could affect conventional hydrological frequency analysis [38,39,40]. On this basis, the Pearson type III (P-III) distribution was used to fit the runoff frequency curve. The distribution parameters were initially estimated using the method of moments and subsequently refined through continuous parameter optimization using a curve-fitting procedure. The root mean square error between the theoretical frequency curve and the empirical frequency points was minimized to determine the optimal parameter combination and calculate characteristic runoff values corresponding to different design frequencies [41]. Based on the fitted P-III distribution, representative design frequencies were selected to characterize wet, dry, and extremely dry hydrological conditions, and the corresponding theoretical runoff values were used to calibrate the water-supply boundaries under these hydrological scenarios. The normal scenario was anchored to the actual hydrological conditions in 2024 to maintain consistency between the hydrological scenario setting and the socioeconomic baseline.

#### 4.3.2. Calibration of Water-Supply Boundaries

Given the strict policy constraints on groundwater extraction in the Shiyang River Basin, including groundwater extraction control and reduction requirements [35,36], groundwater was treated as a relatively stable policy-constrained boundary. Empirical evidence further indicates that the implementation of the Comprehensive Treatment Program of the Shiyang River Basin substantially reduced the groundwater exploitation pressure in the lower basin and contributed to groundwater-level stabilization in Minqin [42]. Therefore, groundwater was not regarded as an unrestricted compensatory source that would automatically increase with declining surface-water availability. Instead, variations in surface runoff were regarded as the primary source of fluctuations in agricultural water supply. This treatment does not imply that actual groundwater abstraction remains physically unchanged among years but rather defines a relatively stable management boundary for the scenario analysis.

Following previous scenario-setting approaches for agricultural water allocation and cropping-structure optimization under representative hydrological years and water-resource uncertainty [18,43], this study defined a dimensionless water regulation coefficient θ. It was calculated as the ratio of the theoretical runoff corresponding to a given design frequency to the multi-year average runoff. This coefficient was used to represent the relative change in the surface water-supply boundary under different hydrological scenarios.

On this basis, a scenario-baseline approach commonly used in hydro-economic modeling was adopted [22]. A decoupled framework of “status quo baseline anchoring–theoretical variation extraction” was used. The actual cropping structure, crop prices, production costs, and water-use control indicators in 2024 were used as the baseline. Socioeconomic parameters, groundwater constraints, and other policy conditions were kept constant. The agricultural water-supply boundary of each county or district was then adjusted according to the water regulation coefficient.

### 4.4. Dynamic Accounting of Crop Water Footprints

The crop water footprint (WF) represents water consumption during crop production [44]. It consists of green, blue, and gray water footprints [45]. The total unit water footprint was calculated as follows:

(1) $${WF}_{ij}={WF}_{blue,ij}+{WF}_{green,ij}+{WF}_{grey,ij}$$

where ${WF}_{ij}$ is the total unit water footprint of crop j in county or district i. ${WF}_{blue,ij}$,$\;{WF}_{green,ij}$ and ${WF}_{grey,ij}$ denote the unit blue, green, and gray water footprint intensities of crop j in county or district i, respectively (m^3^ kg^−1^).

#### 4.4.1. Crop Water-Requirement Accounting

The CROPWAT model was used to calculate the crop water requirement and effective rainfall during the growing period of each crop. The crop water requirement ($ET_{c}$) depends on the crop coefficient ($K_{c}$) and the reference crop evapotranspiration ($ET_{0}$), which can be calculated as follows:

(2) $${ET}_{c}=K_{c}\times {ET}_{0}$$

Reference crop evapotranspiration ($ET_{0}$) was calculated using the FAO-56 Penman–Monteith equation:

(3) $${ET}_{0}=\frac{0.408∆\left(R_{n}-G\right)+\gamma \left(\frac{900}{T+273}\right)u_{2}\left(e_{s}-e_{a}\right)}{∆+\gamma \left(1+0.34\;u_{2}\right)}$$

where ${ET}_{0}$ represents the reference crop evapotranspiration (mm d^−1^); $R_{n}$ is the net radiation at the crop surface (MJ m^−2^ d^−1^); G signifies the soil heat flux density (MJ m^−2^ d^−1^); T is the mean daily air temperature at a height of 2 m (°C); $u_{2}$ denotes the wind speed at a height of 2 m (m s^−1^); $e_{s}-e_{a}$ represents the saturation vapor pressure deficit (kPa); ∆ is the slope of the vapor pressure curve (kPa °C^−1^); and γ is the psychrometric constant (kPa °C^−1^).

Effective rainfall ($P_{e}$) was calculated using the USDA Soil Conservation Service method:

(4) $$P_{e}=\left\{\begin{array}{c} P_{0}\times \frac{\left(125-0.6\times P_{0}\right)}{125},P_{0}\leq \frac{250}{3}\\ \frac{125}{3}\;+\;0.1\;\times \;P_{0},P_{0}\;\geq \frac{250}{3} \end{array}\right.$$

where $P_{0}$ represents precipitation over a 10-day period (mm), and the crop coefficients ($K_{c}$) used for the different crops are listed in Table 4.

#### 4.4.2. Scenario-Based Yield Response

To characterize the effect of changes in agricultural water supply on crop yield, this study introduced the yield response factor from the FAO crop yield–water response framework (Table 4). This coefficient reflects the sensitivity of different crops to water deficit. Considering that the yield reduction caused by water deficit is usually stronger than the yield increase caused by additional water supply, an asymmetric piecewise function was used to describe the yield change in crop in county or district under different hydrological scenarios:

(5) $$Y_{ij}\left(\theta \right)=\left\{\begin{array}{c} Y_{ij,0}\times \left[1-K_{y,j}\times \left(1-\theta \right)\right]\;\;\;\;\;\;\;\;\;\;\;\;\;\;\;\;\;\;\theta \;\leq 1\;\\ Y_{ij,0}\;(1+\delta_{j})\;\;\;\;\;\;\;\;\;\;\;\;\;\;\;\;\;\;\;\;\;\;\;\;\;\;\;\;\;\;\;\;\;\;\;\;\;\;\;\;\theta >1\; \end{array}\right.$$

where $Y_{ij}\left(\theta \right)$ is the yield of crop j in county or district i under hydrological scenario θ. $Y_{ij,0}$ is the baseline-year yield. $K_{y,j}$ is the yield response factor of crop j, which indicates the relative yield reduction under water-deficit conditions [46]. $\delta_{j}$ is the limited yield-increase coefficient under the wet scenario. Based on studies on water-saving irrigation and crop water response in arid areas of Northwest China [47,48], $\delta_{j}$ was set to 0.03 for grain and oilseed crops. It was set to 0.05 for tubers, vegetables, and melons. These coefficients were treated as conservative scenario parameters for representing limited yield gains under wet conditions rather than as locally calibrated physiological constants. Detailed crop-response parameters and scenario-specific county-level yields are provided in Supplementary Tables S9 and S10.

At the county scale, changes in agricultural water supply are mainly transmitted through irrigation water-supply boundaries and quota adjustments. Continuous observations of actual crop evapotranspiration and maximum evapotranspiration are difficult to obtain under different hydrological scenarios. Therefore, the water regulation coefficient θ was used as a scenario variable representing relative water-supply conditions. Under normal and water-deficit scenarios, yield reductions were jointly determined by θ and $K_{y}$, with θ representing relative water-supply conditions and $K_{y}$ characterizing crop-specific sensitivity to water deficit. Under the wet scenario, $\delta_{j}$ was used to limit the increase in crop yield and avoid overestimating the yield gains from additional water supply. This function was used to compare relative yield responses under different hydrological scenarios at the county scale. It was not intended to simulate field-scale crop growth processes.

#### 4.4.3. Scenario-Based Water Footprint Accounting

(1) Dynamic Calibration of Blue and Green Water Footprints

Based on the calculated crop water requirement (${ET}_{c}$) and effective rainfall ($P_{e}$), scenario-dependent crop yield ($Y_{ij}\left(\theta \right)$) and realizable irrigation water availability were incorporated into the calculation of blue and green water footprints under different hydrological scenarios:

(6) $${WF}_{blue,ij}\left(\theta \right)=\frac{10\times max\left(0,{ET}_{c}-P_{e}\right)\times min(1,\theta )}{Y_{ij}\left(\theta \right)}$$

(7) $${WF}_{green,ij}\left(\theta \right)=\frac{10\times min\left({ET}_{c},P_{e}\right)}{Y_{ij}\left(\theta \right)}$$

where 10 is the unit-conversion factor, and min(1,θ) represents the scenario-specific adjustment of realizable blue water application. When θ<1, blue water application per unit area is reduced in proportion to relative water availability, whereas when θ≥1, it is capped at the baseline crop irrigation requirement. Green water is not directly scaled by θ, because the hydrological scenarios represent changes in surface-water availability rather than precipitation.

(2) Gray Water Footprint Accounting

Given the relatively high fertilizer application intensity in agricultural production in the Shiyang River Basin, this study developed a nitrogen-related gray water footprint model based on dynamic yield response to characterize the yield-mediated amplification of pollution loads per unit output under drought conditions [44]:

(8) $${WF}_{grey,ij}\left(\theta \right)=\frac{\alpha \cdot {AR}_{ij}}{\left(C_{max,n}-C_{nat,n}\right)\cdot Y_{ij}\left(\theta \right)}$$

where $AR_{ij}$ denotes the nitrogen application rate per unit area (kg ha^−1^); α represents the nitrogen leaching rate; $C_{max,n}$ and $C_{nat,n}$ are the maximum permissible concentration and the natural background concentration of nitrogen, respectively. The nitrogen application rates for wheat, maize, tubers, vegetables, melons, and oilseeds were 165.15, 260.40, 133.35, 254.70, 240.00, and 147.45 kg ha^−1^, respectively. Except for melons, the crop-specific nitrogen application rates were based on the Gansu provincial averages reported in the National Compilation of Agricultural Product Cost–Benefit Data, whereas the value for melons was set to 240 kg ha^−1^ based on field experiments in arid Northwest China [49,50]. Based on previous studies [44], α, $C_{nat,n}$, and $C_{\max,n}$ were uniformly set to 0.10, 0, and 0.01 kg m^−3^ (10 mg L^−1^), respectively, for all crops and counties.

Nitrogen application rates were maintained at their 2024 baseline values across the four hydrological scenarios to isolate the effects of hydrological variability and yield responses on the gray water footprint. This controlled-management assumption avoids introducing an additional uncertain behavioral response of fertilizer application to water availability. Therefore, hydrological variation was transmitted to the gray water footprint calculation through changes in crop yield, while nitrogen-input and pollutant-related parameters were held constant across scenarios.

(3) Basin-scale water footprint aggregation

Considering differences in crop planting area and yield among counties, the basin-weighted unit water footprint was calculated using crop production as the weight:

(9) $${\bar{WF}}_{c,j}\left(\theta \right)=\frac{\sum_{i=1}^{n}{WF}_{c,ij}\;(\theta )Y_{ij}\;(\theta )X_{ij}}{\sum_{i=1}^{n}Y_{ij}\;(\theta )X_{ij}}$$

The corresponding total water footprint at the basin scale was calculated as follows:

(10) $${TWF}_{c,j}\;\left(\theta \right)=\sum_{i=1}^{n}{WF}_{c,ij}\;\left(\theta \right)Y_{ij}\;\left(\theta \right)X_{ij}$$

where ${\bar{WF}}_{c,j}\left(\theta \right)$ and ${TWF}_{c,j}\;\left(\theta \right)$ are the basin-weighted unit water footprint (m^3^ kg^−1^) and total water footprint (m^3^) of crop j under hydrological scenario θ, respectively. c denotes the blue, green, gray, or total water footprint. ${WF}_{c,ij}\;\left(\theta \right)$, $Y_{ij}\;\left(\theta \right)$ and $X_{ij}$ are the unit water footprint, yield, and planting area of crop j in county or district i, respectively. n is the number of counties or districts included in the study. In the status quo analysis, the planting area was fixed at the actual value in 2024. In the optimization analysis, the optimized planting area under each hydrological scenario was used.

### 4.5. Construction of the Cropping-Structure Optimization Model

#### 4.5.1. Objective Functions

To balance net economic return, resource consumption, and environmental loads, a multi-objective optimization model was constructed. Let $X_{ij}$ denote the planting area (ha) of crop j in county i, and let m denote the number of crop types. The objective functions were formulated as follows:

(1) Maximization of Net Economic Return ($F_{1}$):

(11) $${MaxF}_{1}=\sum_{i=1}^{n}\sum_{j=1}^{m}\left(P_{ij}\times Y_{ij}\left(\theta \right)-C_{ij}\right)\times X_{ij}$$

(2) Minimization of Blue Water Consumption ($F_{2}$):

(12) $${MinF}_{2}=\sum_{i=1}^{n}\sum_{j=1}^{m}{WF}_{blue,ij}\left(\theta \right)\times Y_{ij}\left(\theta \right)\times X_{ij}$$

(3) Minimization of Gray Water Environmental Load ($F_{3}$):

(13) $${MinF}_{3}=\sum_{i=1}^{n}\sum_{j=1}^{m}{WF}_{grey,ij}\left(\theta \right)\times Y_{ij}\left(\theta \right)\times X_{ij}$$

where $F_{1}$ represents the total annual net economic return of the region (CNY); $P_{ij}$ denotes the market price of crop j in county i (CNY kg^−1^); $C_{ij}$ is the comprehensive production cost per unit area (CNY ha^−1^); $F_{2}$ signifies the total regional blue water footprint volume (m^3^); and $F_{3}$ represents the total regional gray water footprint (m^3^). The scenario-specific blue water footprint defined in Equation (6), including the adjustment of realizable irrigation water availability, was consistently used in objective $F_{2}$.

#### 4.5.2. Constraints

(1) Agricultural water-supply boundary constraint:

Based on the calibrated water regulation coefficient, the county-level agricultural water-use control indicators in 2024 were adopted as policy-based upper bounds for agricultural water availability and mapped to the corresponding water-supply boundaries under different hydrological scenarios. To ensure that blue water consumption for crop production in each county or district did not exceed the corresponding scenario-based water-supply boundary, the following constraint was imposed:

(14) $$\sum_{j=1}^{m}{WF}_{blue,ij}\left(\theta \right)\times Y_{ij}\left(\theta \right)\times X_{ij}\leq W_{quota,i}\times \theta$$

where $W_{quota,i}$ represents the baseline-year agricultural water-use control indicator of county or district i (m^3^). In this study, $W_{quota,i}\times \theta$ was used as a scenario-based proxy for the upper agricultural water-supply boundary. The blue water term on the left-hand side of Equation (14) was based on the same scenario-specific blue water formulation as Equation (6).

(2) Crop area constraint:

To account for both the total cultivated-land limit and the practical feasibility of cropping-structure adjustment, and to avoid extreme allocations that are difficult to implement in the short term, the total planted area in each county or district was constrained not to exceed the regional land carrying capacity. Meanwhile, the planting area of each crop was limited to 70–130% of its current area in 2024, based on previous cropping-structure optimization studies [30,51]:

(15) $$\sum_{j=1}^{m}X_{ij}\leq A_{total,i}$$

(16) $$0.7\times X_{ij,0}\leq X_{ij}\leq 1.3\times X_{ij,0}$$

where $A_{total,i}$ denotes the upper limit of the total sown area in county i (ha); and $X_{ij,0}$ is the actual planting area of crop j in county i in the baseline year (ha).

(3) Basic agricultural product supply constraint:

Based on the minimum per capita consumption recommendations in the Chinese Dietary Guidelines 2022 [52], the following constraint was imposed to ensure basic staple-food and nutritional requirements:

(17) $$\sum_{i=1}^{n}\sum_{j\in G_{k}}Y_{ij}\left(\theta \right)\times X_{ij}\geq \sum_{i=1}^{n}{Pop}_{i}\times D_{k,min}$$

where ${Pop}_{i}$ represents the population of county i; $D_{k,min}$ denotes the minimum annual per capita demand for the k-th category of agricultural products (specifically set as: cereals 73.00, tubers 18.25, vegetables 109.50, fruits 73.00, and oilseeds 22.83 kg capita^−1^ year^−1^); and $G_{k}$ signifies the specific set of crops corresponding to the k-th category of agricultural products.

(4) Non-negativity Constraint:

(18) $$X_{ij}\geq 0$$

### 4.6. Model Solving and Scheme Selection

#### 4.6.1. NSGA-III-Based Model Solution

The three-objective cropping-structure optimization model was solved using NSGA-III [53,54] with a population size of 240 and 1000 generations. Simulated binary crossover and polynomial mutation were used with a crossover probability of 0.9, distribution indices of 20, and a mutation probability of 1/30. A total of 153 Das–Dennis reference directions (H = 16) were adopted. Each hydrological scenario was independently optimized five times using fixed random seeds in MATLAB R2025b. Solution repeatability was evaluated using the feasible solution ratio, normalized hypervolume (HV), Spacing, and the coefficient of variation in HV; normalized HV was calculated after min–max normalization using [1.1,1.1,1.1] as the reference point. Detailed information on model parameters, crop economic inputs, water-use constraints, and reproducibility settings is provided in the Supplementary Materials (Tables S11–S16).

Because the model is intended for normative optimization rather than historical prediction, historical cropping patterns were not used as direct validation targets; instead, the observed 2024 cropping structure and actual county-level constraints were used to define the feasible solution space. In addition, an ε-constraint linear programming benchmark was conducted to independently evaluate the NSGA-III solutions. For each selected NSGA-III compromise solution, the corresponding blue and gray water footprint values were imposed as ε constraints, and net economic return was maximized under the same land, water-supply, food-security, and crop-area constraints. The resulting LP solutions were then compared with the corresponding NSGA-III solutions to quantify the relative optimality gap. The detailed benchmark results are provided in Supplementary Table S17.

#### 4.6.2. Entropy-Weighted TOPSIS Scheme Selection

The entropy-weighted TOPSIS method was used to identify the optimal compromise scheme from the Pareto solution set [55]. First, net economic return, blue water consumption, and gray water footprint were normalized, with net economic return treated as a benefit-type indicator and blue water consumption and gray water footprint treated as cost-type indicators. The entropy weights were then determined based on the information dispersion of each indicator. Subsequently, the distances between each candidate scheme and the positive and negative ideal solutions were calculated, and the corresponding relative closeness coefficient was obtained. The scheme with the highest relative closeness coefficient was ultimately selected as the optimal compromise scheme for each hydrological scenario. However, the selected compromise solution may still depend on the resulting objective-weight structure; therefore, it should be interpreted as a data-driven compromise under the entropy-weighting framework rather than as a uniquely weight-independent optimum.

## 5. Conclusions

This study constructed four hydrological scenarios based on the 1995–2024 runoff series and coupled crop yield response, water footprint accounting, and multi-objective optimization to evaluate cropping-structure adjustment in the Shiyang River Basin. The main conclusions are as follows:

(1) The overall direction of cropping-structure optimization remained stable across different hydrological scenarios. The optimized schemes were generally characterized by reductions in wheat, maize, and tuber areas, expansion of melon area, and flexible adjustments in vegetable and oilseed areas in response to water-supply conditions. Although these adjustment directions remained broadly consistent under different crop-area bounds, their stability was partly influenced by the imposed area constraints.
(2) The optimization effects varied markedly across hydrological scenarios. Relative to the corresponding 2024 cropping structure evaluated under the same hydrological conditions, the selected compromise schemes increased net economic return by 14.58–16.23 × 10^8^ CNY across the four scenarios. However, absolute net economic return declined markedly as hydrological conditions deteriorated, from 73.74 × 10^8^ CNY under the wet scenario to 17.95 × 10^8^ CNY under the extremely dry scenario. Meanwhile, the blue water footprint declined from 10.68 × 10^8^ to 7.68 × 10^8^ m^3^, whereas the gray water footprint remained relatively stable at 5.47–5.52 × 10^8^ m^3^ across scenarios. These results indicate that cropping-structure adjustment partially offset hydrologically induced economic losses, although its capacity became increasingly limited under severe water scarcity.
(3) County-level optimization pathways showed clear spatial differentiation. Liangzhou District and Gulang County should prioritize the intensive development of water-efficient, high-value crops, Minqin County should emphasize water-efficient development of specialty melons, Jinchuan District should enhance the flexibility of cropping-structure adjustment, and Yongchang County should prioritize food-production security and drought-resilient production, thereby forming differentiated regulation pathways adapted to county-specific water-resource conditions.

## Figures and Tables

**Table 1:** Calibration of the water regulation coefficient θ under different hydrological scenarios.

Scenario	P (%)	Runoff Q_P_ (10^8^ m^3^)	Regulation Coefficient (θ)
Wet	25	16.11	1.12
Normal (Baseline)	≈46.5	14.44	1.00
Dry	75	12.53	0.87
Extremely Dry	95	10.45	0.72

**Table 2:** Entropy weights of the three objectives under different hydrological scenarios.

Scenario	Net Economic Return	Blue WF	Gray WF
Wet	0.5159	0.2229	0.2612
Normal	0.4957	0.2369	0.2674
Dry	0.4941	0.2332	0.2727
Extremely Dry	0.5080	0.2340	0.2580

**Table 3:** Basin-scale objective responses of the optimal compromise schemes relative to the corresponding same-scenario 2024-structure baselines.

Scenario	2024-Structure Net Return (10^8^ CNY)	Optimized Net Return (10^8^ CNY)	Blue WF (10^8^ m^3^)	Blue WF Reduction (%)	Gray WF (10^8^ m^3^)	Gray WF Reduction (%)
Wet	57.51	73.74	10.68	18.18	5.52	17.31
Normal	50.63	65.21	10.57	19.07	5.47	18.10
Dry	28.55	43.40	9.23	18.75	5.49	17.86
Extremely dry	3.08	17.95	7.68	18.25	5.51	17.56

**Table 4:** Crop coefficients and water response coefficients of major crops.

Crop	K_c,ini_	K_c,mid_	K_c,end_	Ky
Maize	0.23	1.20	0.35	1.25
Wheat	0.23	1.16	0.40	1.15
Vegetables	0.34	1.21	0.70	1.10
Melons	0.29	1.02	0.76	1.10
Tubers	0.24	1.16	0.75	1.10
Oilseeds	0.30	1.13	0.35	0.95

## Data Availability

The data supporting the findings of this study are available in the article and Supplementary Materials. Further inquiries can be directed to the corresponding authors.

## Supplementary Materials

The following supporting information can be downloaded at https://www.mdpi.com/article/10.3390/plants15182863/s1, Table S1: Scenario- and county-specific unit blue water footprints (m^3^ kg^−1^); Table S2: Scenario- and county-specific unit green water footprints (m^3^ kg^−1^); Table S3: Scenario- and county-specific unit gray water footprints (m^3^ kg^−1^); Table S4: Entropy weights and selected compromise-solution objective values; Table S5: Basin-scale comparison of the same-scenario 2024-structure baseline and selected compromise solutions; Table S6: Full optimized planting areas by county, crop, and hydrological scenario (ha); Table S7: Optimized planting-area changes relative to the observed 2024 baseline (%); Table S8: County-level same-scenario baseline and optimized objective values; Table S9: Crop coefficients and scenario-based yield-response parameters; Table S10: Scenario- and county-specific crop yields used in the optimization (kg ha^−1^); Table S11: Reproducibility settings for NSGA-III and solution-quality evaluation; Table S12: Hydrological scenario parameters used in the optimization; Table S13: Observed 2024 baseline planting areas used to define crop-area bounds (ha); Table S14: County-level agricultural water-use control indicators and land carrying capacities; Table S15: County-level minimum agricultural product supply requirements used in the food-security constraints; Table S16: Crop economic inputs and nitrogen application rates; Table S17: ε-constraint linear-programming benchmark of the selected NSGA-III compromise solutions.

## References

[1] Chen J., Zhang C., Guo P. (2022). A Credibility-Based Interval Multi-Objective Crop Area Planning Model for Agricultural and Ecological Management. Agric. Water Manag..

[2] UNESCO (2023). The United Nations World Water Development Report 2023: Partnerships and Cooperation for Water.

[3] Aslam M.F., Masia S., Spano D., Mereu V., Debolini M., Snyder R.L., Borgo A., Trabucco A. (2025). Modelling Crop Water Demand under Climate Change: The Case of Sardinia Region. Irrig. Sci..

[4] Mitter H., Schmid E. (2021). Informing Groundwater Policies in Semi-Arid Agricultural Production Regions under Stochastic Climate Scenario Impacts. Ecol. Econ..

[5] Vallino E., Ridolfi L., Laio F. (2020). Measuring Economic Water Scarcity in Agriculture: A Cross-Country Empirical Investigation. Environ. Sci. Policy.

[6] Yuan Y., Wang J., Gao X., Huang K., Zhao X. (2025). Optimizing Planting Management Practices Considering a Suite of Crop Water Footprint Indicators—A Case-Study of the Fengjiashan Irrigation District. Agric. Water Manag..

[7] Yan G., Jiang L., Liu Y. (2025). Remote Sensing Identification of Major Crops and Trade-off of Water and Land Utilization of Oasis in Altay Prefecture. Land.

[8] Wang J., Xin L., Wang X., Jiang M. (2022). The Impact of Climate Change and Grain Planting Structure Change on Irrigation Water Requirement for Main Grain Crops in Mainland China. Land.

[9] Pereira L.S., Paredes P., López-Urrea R., Hunsaker D.J., Mota M., Shad Z.M. (2021). Standard Single and Basal Crop Coefficients for Vegetable Crops, an Update of FAO56 Crop Water Requirements Approach. Agric. Water Manag..

[10] FAO (2020). The State of Food and Agriculture 2020. Overcoming Water Challenges in Agriculture.

[11] Zhang T., Tan Q., Zhang S., Wang S., Gou T. (2020). A Robust Multi-Objective Model for Supporting Agricultural Water Management with Uncertain Preferences. J. Clean. Prod..

[12] Wang C., Zhang J., Wang T., Zeng B., Wang B., Chen Y., Chen Y. (2025). Intelligent Optimization-Based Decision-Making Framework for Crop Planting Strategy with Total Profit Prediction. Agriculture.

[13] Shahrokhnia M.H., Sepaskhah A.R. (2016). Effects of Irrigation Strategies, Planting Methods and Nitrogen Fertilization on Yield, Water and Nitrogen Efficiencies of Safflower. Agric. Water Manag..

[14] Agrawal P., Sinha J., Jangre N., Kumar F., Kamalkant, Sinha A., Singh A., Banerjee A., Venkatesh A.S., Pasupuleti S. (2025). Developing an Efficient and Optimized Irrigation Plan under Varying Water-Supply Regimes. Ain Shams Eng. J..

[15] Duan Y., Wang W., Zhuo L., Liu Y., Wu P. (2024). Regional Blue and Green Water-Saving Potential and Regulation Paths for Crop Production: A Case Study in the Yellow River Basin. Agric. Water Manag..

[16] Hu M., Wu W., Yu Q., Tang H., Wen Y., Zhao F. (2022). Spatial-Temporal Variations in Green, Blue and Gray Water Footprints of Crops: How Do Socioeconomic Drivers Influence?. Environ. Res. Lett..

[17] Li J., Li L., Zhou D., Ma X., Chen L., Zhang J., Dong Q. (2026). Optimization of Agricultural Planting Structure in the Shiyang River Basin Based on Multi-Objective Optimization. J. Irrig. Drain..

[18] Chen Y., Zhou Y., Fang S., Li M., Wang Y., Cao K. (2022). Crop Pattern Optimization for the Coordination between Economy and Environment Considering Hydrological Uncertainty. Sci. Total Environ..

[19] Zhang Y., Tan Q., Zhang T., Zhang T., Zhang S. (2022). Sustainable Agricultural Water Management Incorporating Inexact Programming and Salinization-Related Grey Water Footprint. J. Contam. Hydrol..

[20] Meng G., Zhu G., Jiao Y., Qiu D., Wang Y., Lu S., Li R., Liu J., Chen L., Wang Q. (2025). Soil Salinity Patterns Reveal Changes in the Water Cycle of Inland River Basins in Arid Zones. Hydrol. Earth Syst. Sci..

[21] Shao Y., Dong Z., Meng J., Wu S., Li Y., Zhu S., Zhang Q., Zheng Z. (2023). Analysis of Runoff Variation and Future Trends in a Changing Environment: Case Study for Shiyanghe River Basin, Northwest China. Sustainability.

[22] Harou J.J., Pulido-Velazquez M., Rosenberg D.E., Medellín-Azuara J., Lund J.R., Howitt R.E. (2009). Hydro-Economic Models: Concepts, Design, Applications, and Future Prospects. J. Hydrol..

[23] Lauffenburger Z.H., Maneta M.P., Cobourn K.M., Jencso K., Chaffin B., Crockett A., Maxwell B., Kimball J.S. (2022). A Hydro-Economic Analysis of End-of-Century Climate Projections on Agricultural Land and Water Use, Production, and Revenues in the U.S. Northern Rockies and Great Plains. J. Hydrol. Reg. Stud..

[24] Jiang Y., Burney J.A. (2025). Crop Water Origins and Hydroclimate Vulnerability of Global Croplands. Nat. Sustain..

[25] Wang X., Jia H., Wang X., Zhang J., Chen F. (2024). Spatial Distribution of the Cropping Pattern Exerts Greater Influence on the Water Footprint Compared to Diversification in Intensive Farmland Landscapes. Land.

[26] Zhao D., Liu W., Gao R., Zhang P., Li M., Wu P., Zhuo L. (2023). Spatiotemporal Evolution of Crop Grey Water Footprint and Associated Water Pollution Levels in Arid Regions of Western China. Agric. Water Manag..

[27] Liu H., Liu X., Zhang T., Du X., Zhao Y., Luo J., Qiu W., Wu S., Liu H. (2025). Nitrogen and Gray Water Footprints of Various Cropping Systems in Irrigation Districts: A Case from Ningxia, China. Water.

[28] Miao B.-L., Liu Y., Fan Y.-B., Niu X.-J., Jiang X.-Y., Tang Z. (2023). Optimization of Agricultural Resource Allocation among Crops: A Portfolio Model Analysis. Land.

[29] Tan Q., Zhang S., Li R. (2017). Optimal Use of Agricultural Water and Land Resources through Reconfiguring Crop Planting Structure under Socioeconomic and Ecological Objectives. Water.

[30] Yan D., Jia Z., Xue J., Sun H., Gui D., Liu Y., Zeng X. (2018). Inter-Regional Coordination to Improve Equality in the Agricultural Virtual Water Trade. Sustainability.

[31] Zhang H., Zhou Q., Zhang C. (2021). Evaluation of Agricultural Water-Saving Effects in the Context of Water Rights Trading: An Empirical Study from China’s Water Rights Pilots. J. Clean. Prod..

[32] Xie S., Zhang J., Li X., Xia X., Chen Z. (2024). The Effect of Agricultural Insurance Participation on Rural Households’ Economic Resilience to Natural Disasters: Evidence from China. J. Clean. Prod..

[33] Ngabire M., Wang T., Xue X., Liao J., Sahbeni G., Huang C., Song X., Duan H., Nyiransengiyumva C. (2022). Synergic Effects of Land-Use Management Systems towards the Reclamation of Aeolian Desertified Land in the Shiyang River Basin. Ecol. Indic..

[34] Li J., Wang J. (2025). Spatiotemporal Evolution Characteristics of the Resilience of Oasis Social–Ecological Systems in the Hexi Corridor. Arid Zone Res..

[35] General Office of the People’s Government of Gansu Province (2014). Measures for the Management of Groundwater Resources in the Shiyang River Basin.

[36] National Development and Reform Commission, Ministry of Water Resources (2007). Key Management Plan for the Shiyang River Basin.

[37] Duan A., Sun J., Liu Y. (2004). Irrigation Water Quotas for Major Crops in Northern China.

[38] Pettitt A.N. (1979). A Non-Parametric Approach to the Change-Point Problem. J. R. Stat. Soc. Ser. C Appl. Stat..

[39] Li S., Qin Y. (2022). Frequency Analysis of the Nonstationary Annual Runoff Series Using the Mechanism-Based Reconstruction Method. Water.

[40] Mann H.B. (1945). Nonparametric Tests against Trend. Econometrica.

[41] Lei G.-J., Wang W.-C., Yin J.-X., Wang H., Xu D.-M., Tian J. (2019). Improved Fuzzy Weighted Optimum Curve-Fitting Method for Estimating the Parameters of a Pearson Type-III Distribution. Hydrol. Sci. J..

[42] Liu M., Nie Z., Liu X., Wang L., Cao L. (2024). Change in Groundwater Table Depth Caused by Natural Change and Human Activities during the Past 40 Years in the Shiyang River Basin, Northwest China. Sci. Total Environ..

[43] Gong X., Zhang H., Ren C., Sun D., Yang J. (2020). Optimization Allocation of Irrigation Water Resources Based on Crop Water Requirement under Considering Effective Precipitation and Uncertainty. Agric. Water Manag..

[44] Hoekstra A., Chapagain A., Aldaya M.M., Mekonnen M.M., Hoekstra A.Y. (2011). The Water Footprint Assessment Manual: Setting the Global Standard.

[45] Tian F., Zhang Y., Lu S. (2020). Spatial-Temporal Dynamics of Cropland Ecosystem Water-Use Efficiency and the Responses to Agricultural Water Management in the Shiyang River Basin, Northwestern China. Agric. Water Manag..

[46] Doorenbos J., Kassam A.H., Bentvelsen C.I.M. (1979). Yield Response to Water.

[47] Peng S., Bian L., Zhu C. (2000). Research and Development of Crop Water Production Functions. Adv. Sci. Technol. Water Resour..

[48] Du T., Kang S. (2011). Water-Saving, Quality-Regulating and High-Efficiency Irrigation for Specialty Cash Crops Based on Water–Quality Response Relationships. J. Hydraul. Eng..

[49] Du S., Ma Z., Xue L. (2016). Effects of Combined Application of Nitrogen, Phosphorus and Potassium on Yield and Quality of Watermelon in Gravel-Mulched Fields. J. Plant Nutr. Fertil..

[50] Xue L., Ma Z., Du S. (2014). Effects of Water–Nitrogen Coupling on Soil Nitrate Nitrogen Transport and Nitrogen Uptake of Melon in an Oasis Irrigation Area. J. Plant Nutr. Fertil..

[51] Hou Y., Liu Y., Xu X., Fan Y., Ma S., Wang S. (2023). Grid-Scale Crop Dynamic Layout Optimization Model Considering Stakeholders’ Cropping Preferences and Practice Behaviours. Ecol. Indic..

[52] Chinese Nutrition Society (2022). Chinese Dietary Guidelines 2022 and Popular Science Edition, 2-Volume Set.

[53] Jain H., Deb K. (2014). An Evolutionary Many-Objective Optimization Algorithm Using Reference-Point Based Nondominated Sorting Approach, Part II: Handling Constraints and Extending to an Adaptive Approach. IEEE Trans. Evol. Comput..

[54] Deb K., Jain H. (2014). An Evolutionary Many-Objective Optimization Algorithm Using Reference-Point-Based Nondominated Sorting Approach, Part I: Solving Problems with Box Constraints. IEEE Trans. Evol. Comput..

[55] Hwang C., Yoon K. (1981). Multiple Attribute Decision Making: Methods and Applications a State-of-the-Art Survey.

