# Supplementary material for: Coupling Hydrological Scenarios and Water Footprints for Multi-Objective Cropping-Structure Optimization in the Middle and Lower Shiyang River Basin
Source: Plants (Basel). 2026 Sep 19;15(18):2863. doi: 10.3390/plants15182863 (PMC13610305; doi:10.3390/plants15182863)
Supplement: Supplementary file 1 [file plants-15-02863-s001.zip › plants-4541048-supplementary.pdf]

Table S1. Scenario- and county-specific unit blue water footprints (m³ kg⁻¹).

Scenario	County/District	Wheat	Maize	Tubers	Vegetables	Melons	Oilseeds
Wet	Liangzhou District	0.5641	0.4126	0.1038	0.0667	0.0429	1.2262
Wet	Minqin County	0.7010	0.6583	0.1086	0.0971	0.0714	0.8320
Wet	Gulang County	0.5951	0.5019	0.1095	0.0600	0.0457	1.5670
Wet	Jinchuan District	0.6621	0.5447	0.1038	0.0933	0.0838	0.8097
Wet	Yongchang County	0.5476	0.4398	0.0857	0.0857	0.1895	0.7282
Normal	Liangzhou District	0.5810	0.4250	0.1090	0.0700	0.0450	1.2630
Normal	Minqin County	0.7220	0.6780	0.1140	0.1020	0.0750	0.8570
Normal	Gulang County	0.6130	0.5170	0.1150	0.0630	0.0480	1.6140
Normal	Jinchuan District	0.6820	0.5610	0.1090	0.0980	0.0880	0.8340
Normal	Yongchang County	0.5640	0.4530	0.0900	0.0900	0.1990	0.7500
Dry	Liangzhou District	0.5943	0.4415	0.1107	0.0711	0.0457	1.2536
Dry	Minqin County	0.7386	0.7043	0.1157	0.1035	0.0761	0.8506
Dry	Gulang County	0.6271	0.5371	0.1167	0.0640	0.0487	1.6020
Dry	Jinchuan District	0.6976	0.5828	0.1107	0.0995	0.0893	0.8278
Dry	Yongchang County	0.5769	0.4706	0.0914	0.0914	0.2020	0.7444
Extremely dry	Liangzhou District	0.6170	0.4708	0.1134	0.0728	0.0468	1.2389
Extremely dry	Minqin County	0.7667	0.7510	0.1186	0.1061	0.0780	0.8407
Extremely dry	Gulang County	0.6510	0.5727	0.1197	0.0655	0.0499	1.5832
Extremely dry	Jinchuan District	0.7242	0.6214	0.1134	0.1020	0.0916	0.8181
Extremely dry	Yongchang County	0.5989	0.5018	0.0936	0.0936	0.2071	0.7357

Note. The unit blue water footprint was calculated using the scenario-dependent water-supply adjustment factor $min(1, \theta)$ *in the formulation.*

Table S2. Scenario- and county-specific unit green water footprints (m³ kg⁻¹).

Scenario	County/District	Wheat	Maize	Tubers	Vegetables	Melons	Oilseeds
Wet	Liangzhou District	0.0981	0.2039	0.0295	0.0314	0.0152	0.3864
Wet	Minqin County	0.0544	0.1350	0.0124	0.0181	0.0086	0.1350
Wet	Gulang County	0.2233	0.3621	0.0486	0.0410	0.0219	0.7126
Wet	Jinchuan District	0.0544	0.1417	0.0124	0.0200	0.0114	0.1146
Wet	Yongchang County	0.1573	0.2252	0.0333	0.0390	0.0819	0.3272
Normal	Liangzhou District	0.1010	0.2100	0.0310	0.0330	0.0160	0.3980
Normal	Minqin County	0.0560	0.1390	0.0130	0.0190	0.0090	0.1390
Normal	Gulang County	0.2300	0.3730	0.0510	0.0430	0.0230	0.7340
Normal	Jinchuan District	0.0560	0.1460	0.0130	0.0210	0.0120	0.1180
Normal	Yongchang County	0.1620	0.2320	0.0350	0.0410	0.0860	0.3370
Dry	Liangzhou District	0.1188	0.2507	0.0362	0.0385	0.0187	0.4541
Dry	Minqin County	0.0658	0.1660	0.0152	0.0222	0.0105	0.1586
Dry	Gulang County	0.2704	0.4454	0.0595	0.0502	0.0268	0.8374

Scenario	County/District	Wheat	Maize	Tubers	Vegetables	Melons	Oilseeds
Dry	Jinchuan District	0.0658	0.1743	0.0152	0.0245	0.0140	0.1346
Dry	Yongchang County	0.1905	0.2770	0.0408	0.0478	0.1004	0.3845
Extremely dry	Liangzhou District	0.1490	0.3231	0.0448	0.0477	0.0231	0.5422
Extremely dry	Minqin County	0.0826	0.2138	0.0188	0.0275	0.0130	0.1894
Extremely dry	Gulang County	0.3392	0.5738	0.0737	0.0621	0.0332	1.0000
Extremely dry	Jinchuan District	0.0826	0.2246	0.0188	0.0303	0.0173	0.1608
Extremely dry	Yongchang County	0.2389	0.3569	0.0506	0.0592	0.1243	0.4591

Note. Unit green water footprint is reported for completeness of the blue-green-gray accounting framework.

Table S3. Scenario- and county-specific unit gray water footprints ($m^3\ kg^{-1}$).

Scenario	County/District	Wheat	Maize	Tubers	Vegetables	Melons	Oilseeds
Wet	Liangzhou District	0.2320	0.2505	0.0359	0.0360	0.0327	0.4939
Wet	Minqin County	0.2143	0.3080	0.0296	0.0427	0.0373	0.2717
Wet	Gulang County	0.2554	0.3713	0.0398	0.0360	0.0352	0.6485
Wet	Jinchuan District	0.2363	0.2856	0.0289	0.0427	0.0482	0.2642
Wet	Yongchang County	0.2308	0.2917	0.0316	0.0430	0.1402	0.3089
Normal	Liangzhou District	0.2390	0.2580	0.0377	0.0378	0.0344	0.5087
Normal	Minqin County	0.2208	0.3172	0.0311	0.0448	0.0392	0.2798
Normal	Gulang County	0.2630	0.3825	0.0417	0.0378	0.0370	0.6680
Normal	Jinchuan District	0.2434	0.2942	0.0304	0.0449	0.0506	0.2721
Normal	Yongchang County	0.2377	0.3004	0.0331	0.0452	0.1472	0.3182
Dry	Liangzhou District	0.2810	0.3080	0.0440	0.0441	0.0401	0.5804
Dry	Minqin County	0.2596	0.3787	0.0363	0.0523	0.0457	0.3193
Dry	Gulang County	0.3092	0.4567	0.0487	0.0441	0.0432	0.7621
Dry	Jinchuan District	0.2862	0.3512	0.0355	0.0523	0.0591	0.3104
Dry	Yongchang County	0.2795	0.3587	0.0387	0.0527	0.1718	0.3630
Extremely dry	Liangzhou District	0.3525	0.3969	0.0545	0.0547	0.0497	0.6931
Extremely dry	Minqin County	0.3256	0.4880	0.0450	0.0648	0.0567	0.3812
Extremely dry	Gulang County	0.3879	0.5884	0.0603	0.0546	0.0535	0.9100
Extremely dry	Jinchuan District	0.3590	0.4526	0.0439	0.0648	0.0731	0.3707
Extremely dry	Yongchang County	0.3507	0.4622	0.0479	0.0653	0.2127	0.4334

Note. Nitrogen application intensity is fixed by crop; therefore, scenario variation in unit gray water footprint arises from the scenario-dependent crop yield in the denominator.

Table S4. Entropy weights and selected compromise-solution objective values.

Scenario	w(Net return)	w(Blue WF)	w(Gray WF)	TOPSIS score	Net return (10^8 CNY)	Blue WF ($10^8\ m^3$)	Gray WF ($10^8\ m^3$)
Wet	0.5159	0.2229	0.2612	0.6304	73.7417	10.6825	5.5249
Normal	0.4957	0.2369	0.2674	0.6155	65.2124	10.5659	5.4718
Dry	0.4941	0.2332	0.2727	0.6256	43.4004	9.2301	5.4876
Extremely dry	0.5080	0.2340	0.2580	0.6496	17.9542	7.6837	5.5074

Note. Entropy weights were calculated separately from each scenario-specific non-dominated solution set and were used in entropy-weighted TOPSIS to identify the reported compromise solution.

Table S5. Basin-scale comparison of the same-scenario 2024-structure baseline and selected compromise solutions.

Scenario	Baseline gross revenue (10^8 CNY)	Baseline production cost (10^8 CNY)	Cost share (%)	Baseline net return (10^8 CNY)	Optimized net return (10^8 CNY)	Δ net return (10^8 CNY)	Baseline blue WF (10^8 m³)	Optimized blue WF (10^8 m³)	Blue WF reduction (10^8 m³)	Baseline gray WF (10^8 m³)	Optimized gray WF (10^8 m³)	Gray WF reduction (10^8 m³)
Wet	158.4192	100.9079	63.70	57.5113	73.7417	16.2304	13.0561	10.6825	2.3736	6.6806	5.5249	1.1557
Normal	151.5386	100.9079	66.59	50.6307	65.2124	14.5817	13.0561	10.5659	2.4902	6.6814	5.4718	1.2096
Dry	129.4604	100.9079	77.94	28.5525	43.4004	14.8478	11.3595	9.2301	2.1294	6.6814	5.4876	1.1939
Extremely dry	103.9856	100.9079	97.04	3.0777	17.9542	14.8765	9.3994	7.6837	1.7156	6.6814	5.5074	1.1740

Note. The unchanged 2024 cropping structure remained feasible under the model constraints in all four hydrological scenarios. Cost share was calculated as baseline production cost divided by baseline gross revenue.

Table S6. Full optimized planting areas by county, crop, and hydrological scenario (ha).

Scenario	County/District	Wheat	Maize	Tubers	Vegetables	Melons	Oilseeds	Total
Wet	Liangzhou District	14,273.60	36,951.52	3,735.45	37,114.61	3,832.21	202.02	96,109.41
Wet	Minqin County	3,644.78	11,883.08	2,873.85	6,719.28	16,349.68	5,097.01	46,567.68
Wet	Gulang County	6,627.70	15,164.13	4,281.37	8,827.01	2,494.84	2,525.53	39,920.57
Wet	Jinchuan District	2,412.98	7,284.97	170.18	5,177.62	328.40	235.56	15,609.72
Wet	Yongchang County	17,661.31	12,662.12	3,619.14	9,580.86	16.73	1,281.86	44,822.02
Normal	Liangzhou District	14,273.04	36,951.50	3,743.54	36,945.23	3,828.85	205.61	95,947.78
Normal	Minqin County	3,649.99	11,883.23	2,894.87	6,719.03	16,310.35	5,099.54	46,557.01
Normal	Gulang County	6,627.29	15,164.05	4,282.34	8,821.99	2,491.41	2,569.02	39,956.11
Normal	Jinchuan District	2,415.58	7,285.03	173.87	3,083.83	318.89	234.78	13,511.98
Normal	Yongchang County	17,661.21	12,660.04	3,619.42	9,722.29	18.84	1,280.91	44,962.70
Dry	Liangzhou District	14,273.00	36,951.33	3,733.93	37,112.71	3,831.89	206.89	96,109.74
Dry	Minqin County	3,646.57	11,883.09	2,877.96	6,792.30	16,349.89	5,762.84	47,312.65
Dry	Gulang County	6,627.32	15,164.78	4,281.28	8,804.87	2,495.07	2,525.66	39,898.98
Dry	Jinchuan District	2,413.53	7,285.10	188.69	3,131.66	328.35	258.62	13,605.94
Dry	Yongchang County	17,661.30	12,660.41	3,619.79	9,572.65	18.82	1,417.79	44,950.77
Extremely dry	Liangzhou District	14,273.01	36,951.63	3,734.05	37,082.17	3,829.26	219.97	96,090.10
Extremely dry	Minqin County	3,644.76	11,883.16	2,873.12	6,714.03	16,350.32	7,380.74	48,846.13
Extremely dry	Gulang County	6,627.33	15,164.03	4,281.32	8,775.26	2,487.37	2,525.11	39,860.42
Extremely dry	Jinchuan District	2,412.92	7,285.39	173.46	3,082.19	329.95	315.94	13,599.84
Extremely dry	Yongchang County	17,661.81	12,660.38	3,618.26	9,586.18	22.64	1,405.73	44,955.00

Note. These are the complete county-crop planting areas of the entropy-weighted TOPSIS compromise solution for each hydrological scenario. The baseline crop-area bounds are 70%-130% of the observed 2024 areas.

Table S7. Optimized planting-area changes relative to the observed 2024 baseline (%).

Scenario	County/District	Wheat	Maize	Tubers	Vegetables	Melons	Oilseeds
Wet	Liangzhou District	-30.00	-30.00	-29.97	29.97	29.99	-29.71
Wet	Minqin County	-30.00	-30.00	-29.95	-29.94	29.99	-29.97

Scenario	County/District	Wheat	Maize	Tubers	Vegetables	Melons	Oilseeds
Wet	Gulang County	-29.99	-30.00	-29.99	29.98	29.82	-29.97
Wet	Jinchuan District	-30.00	-30.00	-29.56	17.68	29.32	-29.47
Wet	Yongchang County	-30.00	-29.99	-29.98	-29.85	-24.89	-29.92
Normal	Liangzhou District	-30.00	-30.00	-29.82	29.37	29.87	-28.46
Normal	Minqin County	-29.90	-30.00	-29.44	-29.94	29.68	-29.93
Normal	Gulang County	-30.00	-30.00	-29.98	29.90	29.64	-28.77
Normal	Jinchuan District	-29.92	-30.00	-28.03	-29.91	25.58	-29.71
Normal	Yongchang County	-30.00	-30.00	-29.98	-28.82	-15.40	-29.98
Dry	Liangzhou District	-30.00	-30.00	-30.00	29.96	29.98	-28.01
Dry	Minqin County	-29.96	-30.00	-29.85	-29.18	29.99	-20.82
Dry	Gulang County	-30.00	-30.00	-30.00	29.65	29.83	-29.97
Dry	Jinchuan District	-29.98	-30.00	-21.90	-28.82	29.30	-22.57
Dry	Yongchang County	-30.00	-30.00	-29.97	-29.91	-15.47	-22.49
Extremely dry	Liangzhou District	-30.00	-30.00	-29.99	29.85	29.89	-23.46
Extremely dry	Minqin County	-30.00	-30.00	-29.97	-29.99	30.00	1.41
Extremely dry	Gulang County	-30.00	-30.00	-29.99	29.22	29.43	-29.98
Extremely dry	Jinchuan District	-30.00	-30.00	-28.21	-29.94	29.93	-5.41
Extremely dry	Yongchang County	-30.00	-30.00	-30.00	-29.82	1.65	-23.15

Note. Percentage change is calculated as 100 × (optimized area - observed 2024 area) / observed 2024 area. Values close to -30% or +30% indicate that the corresponding crop-area bound is active or nearly active.

Table S8. County-level same-scenario baseline and optimized objective values.

Scenario	County/District	Baseline net return (10^8 CNY)	Optimized net return (10^8 CNY)	Δ net return (10^8 CNY)	Baseline blue WF (10^8 m^3)	Optimized blue WF (10^8 m^3)	Blue WF reduction (10^8 m^3)	Baseline gray WF (10^8 m^3)	Optimized gray WF (10^8 m^3)	Gray WF reduction (10^8 m^3)
Wet	Liangzhou District	23.397	33.502	10.104	4.738	4.180	0.558	2.584	2.287	0.297
Wet	Minqin County	21.779	25.111	3.332	2.888	2.369	0.520	1.236	1.046	0.190
Wet	Gulang County	4.598	8.271	3.673	1.828	1.489	0.340	1.074	0.884	0.191
Wet	Jinchuan District	1.693	2.616	0.922	0.958	0.794	0.164	0.454	0.375	0.079
Wet	Yongchang County	6.043	4.243	-1.801	2.643	1.851	0.792	1.332	0.933	0.399
Normal	Liangzhou District	20.652	30.103	9.451	4.737	4.171	0.566	2.585	2.284	0.301
Normal	Minqin County	19.988	23.234	3.246	2.888	2.369	0.520	1.236	1.046	0.190
Normal	Gulang County	3.731	7.278	3.546	1.828	1.490	0.338	1.074	0.884	0.190
Normal	Jinchuan District	1.342	1.067	-0.275	0.958	0.677	0.281	0.454	0.322	0.133
Normal	Yongchang County	4.918	3.531	-1.387	2.643	1.859	0.785	1.332	0.937	0.395
Dry	Liangzhou District	11.784	20.191	8.407	4.122	3.637	0.486	2.585	2.288	0.297
Dry	Minqin County	14.506	17.891	3.385	2.512	2.091	0.422	1.236	1.058	0.178
Dry	Gulang County	0.922	4.233	3.311	1.591	1.295	0.296	1.074	0.883	0.191
Dry	Jinchuan District	0.157	0.242	0.085	0.834	0.593	0.240	0.454	0.324	0.131
Dry	Yongchang County	1.184	0.843	-0.340	2.300	1.615	0.685	1.332	0.935	0.397
Extremely dry	Liangzhou District	1.551	8.588	7.036	3.411	3.008	0.403	2.585	2.287	0.297
Extremely dry	Minqin County	8.180	11.596	3.416	2.079	1.779	0.300	1.236	1.080	0.156

Scenario	County/District	Baseline net return (10^8 CNY)	Optimized net return (10^8 CNY)	Δ net return (10^8 CNY)	Baseline blue WF (10^8 m^3)	Optimized blue WF (10^8 m^3)	Blue WF reduction (10^8 m^3)	Baseline gray WF (10^8 m^3)	Optimized gray WF (10^8 m^3)	Gray WF reduction (10^8 m^3)
Extremely dry	Gulang County	-2.319	0.711	3.030	1.316	1.070	0.246	1.074	0.882	0.192
Extremely dry	Jinchuan District	-1.210	-0.762	0.448	0.690	0.491	0.200	0.454	0.323	0.131
Extremely dry	Yongchang County	-3.125	-2.179	0.946	1.903	1.336	0.567	1.332	0.935	0.397

Note. The baseline for each row is the unchanged observed 2024 cropping structure evaluated under the same hydrological scenario. Positive Δ net return denotes an improvement after optimization; positive water-footprint reduction denotes a decrease relative to the corresponding same-scenario baseline.

Table S9. Crop coefficients and scenario-based yield-response parameters.

Crop	Kc,ini	Kc,mid	Kc,end	Ky	Wet-scenario δ
Wheat	0.23	1.16	0.40	1.15	0.03
Maize	0.23	1.20	0.35	1.25	0.03
Tubers	0.24	1.16	0.75	1.10	0.05
Vegetables	0.34	1.21	0.70	1.10	0.05
Melons	0.29	1.02	0.76	1.10	0.05
Oilseeds	0.30	1.13	0.35	0.95	0.03

Note. Kc values correspond to the regional crop-coefficient source adopted in the manuscript. Ky is the FAO crop yield-response factor. The wet-scenario yield-increase coefficient δ was set to 0.03 for wheat, maize, and oilseeds and to 0.05 for tubers, vegetables, and melons.

Table S10. Scenario- and county-specific crop yields used in the optimization (kg ha⁻¹).

Scenario	County/District	Wheat	Maize	Tubers	Vegetables	Melons	Oilseeds
Wet	Liangzhou District	7117.02	10397.13	37096.92	70685.20	73304.00	2985.35
Wet	Minqin County	7705.62	8455.55	44979.17	59670.56	64276.28	5427.47
Wet	Gulang County	6467.50	7012.39	33546.19	70820.97	68110.31	2273.64
Wet	Jinchuan District	6987.67	9117.62	46075.47	59624.93	49795.92	5582.04
Wet	Yongchang County	7155.02	8927.78	42259.09	59206.64	17117.52	4773.63
Normal	Liangzhou District	6909.73	10094.30	35330.40	67319.24	69813.33	2898.40
Normal	Minqin County	7481.18	8209.27	42837.30	56829.10	61215.50	5269.39
Normal	Gulang County	6279.13	6808.15	31948.75	67448.54	64866.96	2207.42
Normal	Jinchuan District	6784.15	8852.06	43881.40	56785.65	47424.69	5419.46
Normal	Yongchang County	6946.62	8667.75	40246.75	56387.28	16302.40	4634.59
Dry	Liangzhou District	5876.73	8453.98	30278.15	57692.59	59830.02	2540.45
Dry	Minqin County	6362.74	6875.26	36711.57	48702.54	52461.68	4618.62
Dry	Gulang County	5340.40	5701.83	27380.08	57803.40	55590.98	1934.80
Dry	Jinchuan District	5769.92	7413.60	37606.36	48665.30	40642.96	4750.16
Dry	Yongchang County	5908.10	7259.24	34491.46	48323.90	13971.16	4062.22
Extremely dry	Liangzhou District	4684.80	6561.29	24448.64	46584.91	48310.82	2127.43
Extremely dry	Minqin County	5072.24	5336.03	29643.41	39325.74	42361.13	3867.73
Extremely dry	Gulang County	4257.25	4425.30	22108.53	46674.39	44887.94	1620.25
Extremely dry	Jinchuan District	4599.65	5753.84	30365.93	39295.67	32817.89	3977.88
Extremely dry	Yongchang County	4709.81	5634.04	27850.75	39020.00	11281.26	3401.79

Note. Values are scenario-specific yields used to calculate economic returns, food-supply constraints, and unit water footprints. The normal scenario uses the observed 2024 baseline yields.

Table S11. Reproducibility settings for NSGA-III and solution-quality evaluation.

Item	Setting
Number of objectives	3
Decision variables	30 county-crop planting-area variables (5 counties × 6 crops)
Population size	240
Maximum generations	1,000
Crossover operator	Simulated binary crossover (SBX)
Crossover probability	0.9
SBX distribution index (ηc)	20
Mutation operator	Polynomial mutation
Mutation probability	1/nvar = 1/30
Mutation distribution index (ηm)	20
Reference directions	Das-Dennis, H = 16; 153 directions
Constraint handling	Constraint-domination
Independent runs	5 per hydrological scenario
Software	Custom MATLAB implementation
Random seeds	Fixed scenario-specific seeds for the stability evaluation (seed = 1000s + r; s = scenario index, r = run index)
HV normalization	Min-max normalization
Normalized HV reference point	[1.1, 1.1, 1.1]

Note. The hypervolume reference point is used only for post-optimization HV calculation and does not affect the optimization results.

Table S12. Hydrological scenario parameters used in the optimization.

Scenario	Scenario basis	Runoff (10^8 m³)	θ
Wet	P = 25%	16.11	1.12
Normal	Observed 2024 baseline	14.44	1.00
Dry	P = 75%	12.53	0.87
Extremely dry	P = 95%	10.45	0.72

Note. θ is the scenario-specific water-regulation coefficient used to characterize relative agricultural water-supply conditions.

Table S13. Observed 2024 baseline planting areas used to define crop-area bounds (ha).

County/District	Wheat	Maize	Tubers	Vegetables	Melons	Oilseeds	Total
Liangzhou District	20,389.93	52,787.60	5,333.93	28,556.93	2,948.13	287.40	110,303.92
Minqin County	5,206.67	16,975.80	4,102.73	9,590.33	12,577.47	7,278.07	55,731.07
Gulang County	9,467.40	21,662.87	6,115.73	6,791.13	1,921.73	3,606.47	49,565.33
Jinchuan District	3,446.99	10,407.08	241.60	4,399.62	253.94	334.00	19,083.23
Yongchang County	25,230.29	18,085.75	5,168.86	13,658.56	22.27	1,829.27	63,995.00

Note. For the baseline optimization, each county-crop planting area was constrained to 70%–130% of its observed 2024 value.

Table S14. County-level agricultural water-use control indicators and land carrying capacities.

County/District	Agricultural water-use control indicator (10^8 m³)	Agricultural water-use control indicator (m³)	Land carrying capacity, Amax (ha)
Liangzhou District	7.20	720,000,000	114,881.67
Minqin County	4.78	478,000,000	68,363.00
Gulang County	2.53	253,000,000	66,277.40

County/District	Agricultural water-use control indicator (10^8 m³)	Agricultural water-use control indicator (m³)	Land carrying capacity, Amax (ha)
Jinchuan District	1.04	104,000,000	21,111.46
Yongchang County	3.76	376,000,000	68,973.54

Note: County-level agricultural water-use control indicators were compiled from official water-resource management documents and converted into upper-bound constraints for agricultural water consumption in the optimization model.

Table S15. County-level minimum agricultural product supply requirements used in the food-security constraints.

County/District	Population	Grain: wheat + maize (kg yr⁻¹)	Tubers (kg yr⁻¹)	Vegetables (kg yr⁻¹)	Melons/fruits (kg yr⁻¹)	Oilseeds (kg yr⁻¹)
Liangzhou District	874,900	63,867,700	15,966,925	95,801,550	63,867,700	19,973,967
Minqin County	169,500	12,373,500	3,093,375	18,560,250	12,373,500	3,869,685
Gulang County	233,900	17,074,700	4,268,675	25,612,050	17,074,700	5,339,937
Jinchuan District	262,300	19,147,900	4,786,975	28,721,850	19,147,900	5,988,309
Yongchang County	168,200	12,278,600	3,069,650	18,417,900	12,278,600	3,840,006

Note. County-level requirements were calculated from county populations and the minimum per capita demand of each product group and were summed across counties to form the basin-scale food-security constraints in Equation (17).

Table S16. Crop economic inputs and nitrogen application rates.

Crop	Production cost (CNY ha⁻¹)	Price (CNY kg⁻¹)	Nitrogen application rate (kg N ha⁻¹)
Wheat	19,975.65	2.30	165.15
Maize	29,645.10	1.98	260.40
Tubers	29,613.45	0.98	133.35
Vegetables	61,447.50	2.06	254.70
Melons	30,000.00	2.50	240.00
Oilseeds	17,925.00	6.24	147.45

Note. Crop prices and production costs were fixed at the 2024 level. Nitrogen application rates were fixed across hydrological scenarios. In gray-water accounting, the nitrogen leaching fraction $\alpha = 0.10$, $C_{max} = 0.01$ kg m⁻³, and $C_{nat} = 0$.

Table S17. ε-constraint linear-programming benchmark of the selected NSGA-III compromise solutions.

Scenario	NSGA-III net return (10^8 CNY)	LP net return (10^8 CNY)	Optimality gap (%)	NSGA-III blue WF (10^8 m³)	NSGA-III gray WF (10^8 m³)
Wet	73.7417	73.7550	0.0180	10.6825	5.5249
Normal	65.2124	65.3242	0.1711	10.5659	5.4718
Dry	43.4004	43.4274	0.0622	9.2301	5.4876
Extremely dry	17.9542	17.9858	0.1760	7.6837	5.5074

Note. For each NSGA-III compromise solution, its blue- and gray-water objective values were imposed as ε constraints and net economic return was maximized by linear programming. Across all 20 repeated-run compromise points, the mean optimality gap was 0.0948% and the maximum was 0.1760%.
